# Evaluating the Efficacy of a Serious Game to Deliver Health Education About Invasive Meningococcal Disease: Clustered Randomized Controlled Equivalence Trial

**DOI:** 10.2196/60755

**Published:** 2025-02-11

**Authors:** Lauren Bloomfield, Julie Boston, Martin Masek, Lesley Andrew, Donna Barwood, Amanda Devine

**Affiliations:** 1 School of Medicine The University of Notre Dame Australia Fremantle Australia; 2 Edith Cowan University Joondalup Australia

**Keywords:** serious games, meningococcal disease, immersive digital applications, health promotion, gaming, meningitis, infection, bacteria, contagious, infectious, immersive, education, mHealth, mobile health, applications, youth, adolescents

## Abstract

**Background:**

Invasive meningococcal disease (IMD) is a serious, vaccine-preventable infectious disease that can be life-threatening. Teaching adolescents about the early detection and prevention of IMD can be challenging in a school environment, with educators reporting they lack confidence or expertise to cover this in the classroom environment. Professional guest educators are an alternative to cover specialist topics such as IMD; however, time and resourcing constraints can mean that these educators are not always available. Serious games may be an alternative to face-to-face education, where complex health information may be delivered via self-directed gameplay.

**Objective:**

This study aims to develop a serious game that can replace a face-to-face educator in a classroom setting to educate adolescents aged 12 years to 15 years. This study evaluates the efficacy of the Meningococcal Immunisation Awareness, Prevention and Protection app (MIApp), a serious game designed to replicate the information provided in a 30-minute face-to-face presentation provided by a trained educator.

**Methods:**

This clustered, randomized controlled equivalence trial involved students (Years 7-10) from 6 secondary schools across metropolitan Western Australia who completed pre- and postintervention questionnaires with a follow-up at 3 months postintervention to measure the primary outcome of IMD knowledge acquisition following this self-guided intervention. The findings were compared with changes in an active control (comparison) group who received an in-class educational presentation about IMD transmission and protection. A questionnaire was developed to assess 9 key areas of knowledge. Median scores for knowledge pre- and postintervention were collected from a self-administered assessment of this questionnaire and, at 3 months postintervention, were compared between groups. A knowledge score of +/–2/16 was determined a priori to meet the criteria for equivalence. Participants who used MIApp were also asked a series of questions to assess the enjoyment of and engagement with the game.

**Results:**

Of the 788 participating students, the median postintervention correct score in both the MIApp and control cohorts was 14/16 (87.5% correct responses), compared with the median pre-intervention correct score of 6/16 (37.5% correct responses), representing a significant (*P*<.001) increase in IMD knowledge in both groups. Improvements were retained in both groups 3 months after the initial intervention (median correct score: 11/16 in the intervention group; 12/16 in the control group; *P*=.86), demonstrating the efficacy of MIApp to deliver health education about IMD transmission and protection, although response rates in the follow-up cohort were low (255/788, 32.4%).

**Conclusions:**

MIApp met the predetermined threshold for equivalence, demonstrating similar improvements in knowledge posttrial and at the 3-month follow-up. Participating adolescents considered the MIApp game more enjoyable than a presentation, with equivalent improvements in knowledge. Serious games could represent a constructive tool to help teachers impart specialized health education.

## Introduction

An estimated 94% of Australian teenagers are smartphone users, with research showing 82% of teenagers frequently use these devices for digital gaming [1]. The emergence of digital technologies has led to a generation with new ways of thinking, learning, and interacting with each other [2]. Game-based learning occurs through “an environment where game content and gameplay enhance knowledge and skill acquisition, and where game activities involve problem solving spaces and challenges that provide players/learners with a sense of achievement” [3]. In the school environment, digital games are increasingly being considered a constructive learning tool to captivate and intrinsically motivate adolescents’ engagement [4-6]. The concept of “serious games” generally refers to those digital games in which the intention is to provide meaningful learning while simulating real-world scenarios that are typically aligned with educational standards [7-9].

Research suggests serious games can promote active learning, build knowledge, engage and motivate students, support higher-level thinking, and increase learner independence [10]. Studies have also demonstrated the attractiveness of digital games as a learning environment to support social, emotional, and cognitive outcomes in young people [11-13], with evidence illustrating their influence on the long-term retention of knowledge [4,11]. Serious games appear to be well suited to specific mandated curriculum learning areas like science, technology, engineering, and mathematics [14-16] and offer additional learning opportunities to engage adolescents in new emerging fields of study such as health promotion [17,18].

Given their growing appeal, serious games are increasingly being explored as a novel alternative to the traditional classroom delivery of health promotion messages to adolescents [2,5,19,20]. Serious games for health promotion can stimulate real-life situations, providing adolescents with explorative learning environments [21], and can be adapted to the developmental stage, educational level, and personal interest, allowing the player to self-manage their educational goals [22].

Moreover, serious games can offer support for the delivery of health education around issues that may not be easy for teachers to address due to time, stigma, and their own lack of knowledge [22,23]. In addition, a lack of resources for expert presenters to deliver face-to-face content across large geographical areas means that regional and remote areas are less likely to receive such interventions, furthering the inequities between metropolitan and rural populations. A large body of research exists to illustrate how serious games can be designed specifically for topics such as nutrition [14,24,25], sexual health [26,27], substance use [28], and mental health [29], among others. This ranges from understanding strategies that can be used to explain complex concepts to pedagogical approaches that capture the social and cultural nuances of communities or age-related groups. However, despite 2 meta-analyses suggesting serious games represent promising learning tools [5,6], limited and inconsistent evidence of serious game efficacy in delivering health education content remains an issue [27,30].

For digital games to become a key tool for the support of traditional modalities of teaching and learning, there is a need to evaluate the efficacy of these games. The challenge for teachers is to ensure the serious game is curriculum-aligned, fit-for-purpose, and effective as a resource to meet the needs of a range of student ages and settings and that it can extend or complement learning. Developing serious games for health education, however, is time-consuming and challenging. It requires multidisciplinary team members to address stated objectives, encompassing elements of design, content, format, and pedagogy to the psychology of behavioral change [31]. Adapting complex health information for suitability with younger audiences within an educational environment can pose several further challenges for game developers. These challenges include maintaining player engagement and ensuring the game effectively reflects national curriculum standards and learning goals and outcomes.

The Meningococcal Immunisation Awareness, Prevention and Protection app (MIApp) is a serious game developed in Western Australia (WA) to deliver education via a digital platform about the transmission of meningococci and protection targeted at adolescents aged 12 years to 15 years. Invasive meningococcal disease (IMD), caused by *Neisseria meningitidis* bacteria, is a serious, vaccine-preventable infectious disease that is rare but life-threatening [32]. IMD can result in ongoing disability ranging from major neurological complications, including hearing loss, cognitive deficit, and seizure, to severe non-neurological effects, such as the amputation of a limb and death [33]. Adolescents are at an increased risk of meningococcal carriage, a precursor for the development of invasive disease [34]. Multilevel public health measures to reduce the risk of IMD include vaccination; minimization of behaviors known to increase transmission, such as intimate kissing and openly sneezing and coughing; and education for the early identification and antibiotic administration for suspected cases [35]. Anecdotal experience of educators suggests teachers in WA are hesitant to introduce the subject of IMD to students without adequate support from a reliable information source. Current educational strategies for IMD are limited by the availability of (often volunteer) staff to provide face-to-face presentations to school-aged children and by the coordination of organizing these, especially in rural and remote areas.

The primary aim of the study was to evaluate the efficacy of MIApp, a serious game that delivers health education for adolescents about IMD, compared with that of a face-to-face presentation.

The associated objectives were as follows: (1) evaluate the efficacy of the serious game MIApp on knowledge about meningococcal carriage, disease signs and symptoms, and vaccination among adolescents; (2) assess the acceptability of, usability of, and overall student experience with MIApp. The overall purpose of the investigation was to determine whether the delivery via an iPad of a fun, engaging, age-appropriate, self-navigating MIApp could be used as a valuable pedagogical tool to help educators better engage with young people around health issues they may find challenging to address. The secondary aim was to assess the retention of knowledge 3 months postintervention.

## Methods

### Design

The development of the educational MIApp was part of a broader initiative funded by Lotterywest, the WA Department of Health, the Amanda Young Foundation (AYF), and Edith Cowan University (ECU) between 2019 and 2022. The project aimed to raise awareness and promote IMD prevention among adolescents, a high-risk group. MIApp, a serious game, integrates thoughtfully chosen gamification mechanics and educational components. The app was developed using an iterative co-design approach, with collaboration among the funding partners and a steering committee of experts in IMD, public health, serious game development, and learning design, as well as input from high school students and end users. The app was developed with the education team of the AYF and was designed to reflect the information in the current face-to-face presentation. Health Promotion Officers developed these resources and designed them to deliver key health messages at an age-appropriate level.

The resulting narrative emerged as an overworld that brings to life the reality of meningococcal infection through interactive gameplay where the players are tasked with solving the source of IMD infection. Through MIApp, the students are introduced to the content including communicable disease, IMD, the concept of “healthy carriers,” signs and symptoms of infection, and preventive health care strategies (“ways” to be safe and remain safe). An extended MIApp user guide outlining all the game's features is available from the AYF website [36] [Multimedia Appendix 6]. Briefly, players collect items while solving the source of the meningococcal infection to increase their work experience points [Multimedia Appendices 2-4]. They interact with the dog character Buddy, who in real life was the pet of Amanda Young. Amanda tragically passed away at 18 years old from IMD, and her legacy is honored through the AYF. Pilot testing took place across 2 sites, involving students from years 7 through 10, with 68 pre- and postsurvey dyads included in the pilot study analysis. Insights from the pilot study guided adjustments to the gameplay and survey tools, leading to the formal evaluation of MIApp. These data were not included in the trial analysis.

Following the launch of MIApp in mid-2021, formal evaluation began in 2021 and was completed in 2022. We compared 2 education delivery models in the study: the intervention group, who used MIApp [Multimedia Appendices 2-4], and the active control (comparison) group, who received an in-class educational presentation [Multimedia Appendix 5]. This in-class presentation covered the same key messages and concepts on which MIApp was based. However, the students in the comparison group listened to a lecture from a qualified AYF presenter while viewing a PowerPoint presentation, followed by a classroom question and answer session. Participants played MIApp independently, without further instruction from the researchers.

Both models of delivery took approximately 50 minutes. Pre- and postintervention questionnaires were conducted to examine student responses to 9 key knowledge areas of IMD (see Textbox 1). The MIApp research design was a cluster randomized controlled trial, following data analysis from the pilot. Students were recruited across 4 year groups from a range of secondary schools. Recruitment and initial pre-post testing occurred between April 2021 and March 2021, with follow-up surveys sent 3 months after the school visit. The number of participating schools recruited was adjusted depending on the number of enrolled students per school and, consequently, the consent rate at each site, with recruitment ceasing when the prespecified sample size was reached.

**Key 9 knowledge areas that were assessed before and after the intervention.**

Key knowledge areas to be assessed 

1. Meningococcal infection is life-threatening but rare
2. Early signs and symptoms, progression of illness
3. Common symptoms of invasive meningococcal disease to look out for
4. What to do if you are experiencing symptoms
5. The importance of vaccination as a prevention tool
6. Which strains are covered by vaccination
7. The significance of “healthy carriers” in disease transmission
8. How the bacteria spread and behaviors that can result in transmission
9. The importance of avoiding behaviors that could spread the bacteria

### Participants

Adolescents, being at increased risk for meningococcal carriage, were the target demographic for this study. Participants were high school students in year levels 7, 8, 9, and 10; aged 12 years to 15 years; and in the metropolitan area of Perth, WA. Schools were purposely selected using an online directory of the School Index of Community Socio-Educational Advantage (ICSEA) [37]. The ICSEA is a scale of socioeducational advantage computed for each school, allowing comparisons between schools based on the level of educational advantage or disadvantage that students bring to their academic studies. School facilities, resources, and staffing do not influence ICSEA. Sampling and recruitment aimed to acquire an equal proportion of male and female students between years 7 and 10 and across the ICSEA school values in the intervention and control groups.

The sample size calculation was based on an expected cluster size of 100 participants, with an intracluster correlation coefficient of 0.02 and an anticipated effect size of 0.4 (Cohen *d*). The design effect was calculated as 1 + (expected average cluster size – 1) × intracluster correlation coefficient. This resulted in a design effect of 2.98. After adjusting for clustering, an adequate sample size of 201 participants was required. To account for an expected 20% attrition, we aimed for an initial total sample size of 750 participants, leading to an expected final sample size of 600.

The effect size of 0.4 was selected as a moderate expected difference between the intervention and control arms, given the nature of the intervention and previous literature on similar studies. The design effect calculation and the adjustment for clustering follow standard methods for cluster randomized trials as described by Donner and Klar [38]. Cohen *d* was used during the design phase to guide the sample size estimation, though nonparametric methods were used in the analysis phase due to the skewness of the data.

A total of 6 secondary schools across metropolitan Perth, WA, gave consent and participated in the research study. Parent and student information sheets informed participants that the trial would compare MIApp with a face-to-face presentation. The consent process was undertaken by the schools, with the research team only engaging with students who had returned consent. All students who returned consent were eligible to participate in the study. Those who did not return consent participated in a different teacher-led activity chosen at the school’s discretion. It was assumed that all students had a base level of computer literacy suitable for participation in the trial.

Due to mandatory lockdowns and COVID-19 rules, the study team could not travel to regional areas within the time limit of the study, and consequently, only metropolitan schools were included. Recruited schools were asked to provide an aggregate number of classes per year group (years 7, 8, 9, and 10) and students per class who were eligible to participate. Despite differences in student ICSEA scores between the MIApp and control groups, with the MIApp group having more significant variation in socioeducational backgrounds, the median was the same for each group.

### Randomization Procedure

This was a clustered randomized controlled equivalence trial. Randomization was performed at a class level within each school. Following school enrollment, each site provided a class list to the research team indicating the number of classes available for participation at each level. Paper consent was collected by the school, who determined the day on which students were eligible to participate based on the provision of consent. Classes within each school were given a unique identifier and stratified by year level. These were then stratified by class level and randomly allocated to receive the intervention or control in sequential order by research team members with no affiliation to the school or knowledge of the class composition. Due to the nature of the intervention, blinding or allocation concealment was not applied. The arm of participation was recorded in the data as a numerical code (1, 2) that was not revealed to the analyst during the statistical analysis before preparing the data for reporting.

### Instrumentation

All participating students were required to complete pre- and postintervention questionnaires designed to measure the primary outcome: change in knowledge of IMD. To assess the 9 key knowledge areas, 16 questions were developed (Multimedia Appendix 1). These included questions on the symptoms of IMD, where it lives in and on the body, how to prevent transmission, and what to do if you suspect you or someone you know may have IMD (Textbox 1). Participants developed a unique identifier based on elements of their name and date of birth. The participants in the intervention group were also asked to rank on a 5-point scale a series of questions about the study’s secondary outcomes: the participants’ perception of usability and engagement with the game and, more generally, about the delivery of educational messages using serious games as a platform [39]. Data on knowledge acquisition, timing, and behavior were also obtained to gain further insight into the overall usability, learnability, and experience of the MIApp game.

The validity and reliability of the research instrument were established in several ways during the project. First, the instrument was closely examined by the research team and the steering committee to ensure alignment with formative research, curriculum text, clarity, and ease of language for the age group and the functioning and facilities within the instrument, such as response mechanisms. The instrument was then pilot tested for the construct validity of the topics included. Minor revisions were made to discordant wording. Test-retest reliability was not performed for the questionnaire items.

### Procedures

Following agreement to participate in the MIApp study and written informed consent provided by the school principal, schools were asked to provide an aggregate number of classes per year group (years 7, 8, 9, and 10) and students per class eligible to participate. A parent or carer and student (participant) information sheet and consent form were disseminated by the schools before the provision of consent and content delivery. This information sheet outlined the rationale for the study, the risks and benefits to participants, and the proposed activity undertaken as part of the research.

The educational content for the intervention and control groups was based on the AYF guidelines to support safer, healthier, and more physically active living by young Australians by creating meaningful, valuable, and strengths-based learning focused on IMD awareness and prevention. They reflect WA's Healthy and Physical Education (HPE) learning outcomes via the WA P-10 Syllabus for HPE and the Australian Curriculum for HPE [40]. The intervention, MIApp, is a new software application developed by ECU. MIApp is a self-directed, serious game designed to be completed in 30 minutes. It targets students in years 7 through 10 to raise awareness and bring to life the realities of meningococcal infection. At the Detect and Protect Agency, players investigate a case to solve the source of a mystery infection. Players engage with the learning content and key messages delivered in the AYF presentation through a combination of games, quizzes, simulations, and interactive activities. The control group received a face-to-face educational presentation delivered by a presenter from the AYF. This is the primary mode of education developed by the AYF and includes a secondary school PowerPoint presentation adapted to fit the allocated research time of 30 minutes. The presentation delivers 9 key messages about IMD including prevention and identification of symptoms and preventative health care strategies (see Textbox 1). Embedded in the presentation is a short interactive quiz and a video highlighting real-life stories of young people who have caught and survived the disease.

Classes within each participating school were randomized to the intervention or control arm stratified by year group, with one-half of the classes at each school receiving either the app or the face-to-face presentation. At the discretion of individual schools, classrooms or locations were set up for the research trials. A requirement was that rooms be separate, so no interference was experienced with either mode of delivery. All questionnaires were completed by the participants using the Qualtrics survey platform [41] on an iPad during the initial onsite visit and via an emailed link for the 3-month follow-up.

Tablets were distributed to all the participants, followed by a briefing on the scope of the research and instructions for the session. Each session (control and intervention) took 50 minutes comprised of 5 minutes to 10 minutes for the pre-intervention questionnaire, 30 minutes for either the intervention (MIApp) or control (presentation), and a further 5 minutes to 10 minutes for the postintervention questionnaire. There was limited interaction between the researchers and participants in order to mirror the use in a classroom environment. Participants were first asked to create a unique identifier that could be used to link the questionnaire data collected before and after their session and again at the 3-month follow-up. The identifier used the first 2 letters of their first name, the day of their date of birth, and the last 2 letters of their last name. MIApp cohort participants also used this unique identifier to link survey responses to in-app data gathered by MIApp. Participants were not required to remember the username they created for the follow-up survey, and no identifying information about the student was collected by the app.

### Data Analysis

Statistical analysis was conducted using R version 4.0.3 [42]. “Differences in correct” scores of the 16 pre and postintervention questions were used to compare the MIApp and control cohorts (Multimedia Appendix 1). Comparative subanalysis of the differences was conducted by age and sex to assess differences in median score improvement between groups. The Kruskal-Wallis test was used for continuous variables [43]. Differences in the proportions of the baseline median included a Pearson chi-square test for categorical variables. Statistically significant differences were considered at *P*<.05. Statistical equivalence testing was also performed using the method by Rusticus and Lovato [44]. First, 95% CIs were calculated using Games-Howell post hoc tests (which account for unequal group sizes and violations in homogeneity of variance). The equivalence interval was defined as ±2 points in the pre-post score difference, which we considered a priori to represent a similar level of improvement. Data were analyzed using intention-to-treat analysis, whereby all participants were analyzed according to the intervention they received on the day of the trial.

### Ethics Approval

Ethics approval for the study was obtained from the Human Research Ethics Committee at ECU (reference: 2019-00736-BLOOMFIELD). The parents of all participants were provided with participant information and a consent form outlining the rationale for the trial and the activities of the intervention and control groups.

### Trial Registration

As this was not clinical research, it was not registered on the Australian New Zealand Clinical Trials Registry. A protocol was not published prior to commencement.

## Results

**Figure 1:**
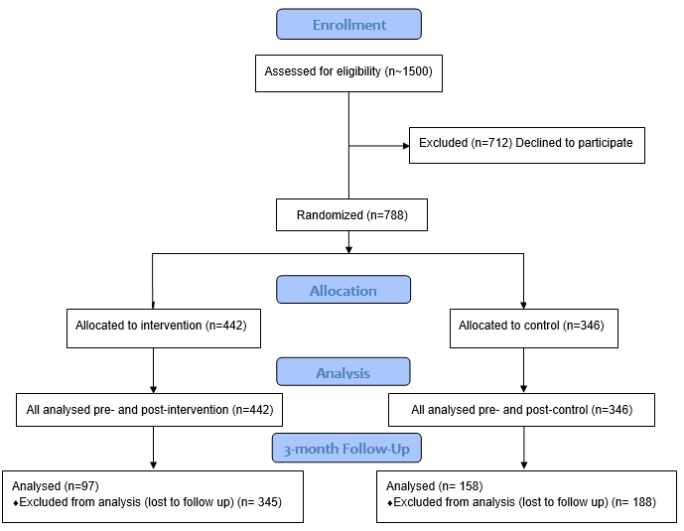
CONSORT Flow Diagram.

Approximately 1500 students were invited to participate in the trial by the 6 schools invited to participate. A total of 788 participants from the 6 trial schools consented and completed the pre- and postintervention questionnaires and were eligible for inclusion in the primary analysis: 442 in the intervention arm and 346 in the control arm (Figure 1). Although different demographic patterns were observed across the 6 participating schools, approximately one-half of the students were in year 7 (397/788, 50.4%), were female (397/788, 50.4%), and self-reported not knowing anything about meningococcal disease at the start of the trial (395/788, 50.1%) (Table 1). The demographic and self-reported pre-knowledge characteristics of the study population by group are shown in Table 2. There were no statistically significant differences between the groups, which suggests a good balance of these properties following group randomization.

**Table 1:** Demographics of the participants.

Demographics		School 1 (n=173), n (%)	School 2 (n=56), n (%)	School 3 (n=138), n (%)	School 4 (n=198), n (%)	School 5 (n=214), n (%)	School 6 (n=9), n (%)	Total sample (N=788), n (%)
ICSEA^a^		1100	1013	1032	1110	1120	890	—^b^
**Year**								
	7	111 (64.2)	26 (46.4)	0 (0)	46 (23.2)	214 (100)	0 (0)	397 (50.4)
	8	62 (35.8)	5 (8.9)	0 (0)	31 (15.7)	0 (0)	6 (66.7)	104 (13.2)
	9	0 (0)	25 (44.6)	138 (100)	24 (12.1)	0 (0)	3 (33.3)	190 (24.1)
	10	0 (0)	0 (0)	0 (0)	97 (49)	0 (0)	0 (0)	97 (12.3)
**Gender**								
	Male	67 (38.7)	31 (55.4)	76 (55.1)	93 (47)	91 (42.5)	3 (33.3)	361 (45.8)
	Female	102 (59)	21 (37.5)	56 (40.6)	97 (49)	116 (54.2)	5 (55.6)	397 (50.4)
	Other	4 (2.3)	4 (7.1)	6 (4.3)	8 (4)	7 (3.3)	1 (11.1)	30 (3.8)

^a^ICSEA: Index of Community Socio-Educational Advantage.

^b^Not applicable.

**Table 2:** Demographic characteristics and self-reported preknowledge of meningococcal disease in the Meningococcal Immunisation Awareness, Prevention and Protection app (MIApp) group versus the control presentation group.

Demographic characteristics and preknowledge			MIApp (n=442)		Control (n=346)		Total sample (n=788)		*P* value^a^	
**Year, n (%)**										.19
	7	215 (48.6)		182 (52.6)		397 (50.4)				
	8	60 (13.6)		44 (12.7)		104 (13.2)				
	9	118 (26.7)		72 (20.8)		190 (24.1)				
	10	49 (11.1)		48 (13.9)		97 (12.3)				
**Gender, n (%)**										.94
	Male	200 (45.2)		161 (46.5)		361 (45.8)				
	Female	225 (50.9)		172 (49.7)		397 (50.4)				
	Other	17 (3.8)		13 (3.8)		30 (3.8)				
**Baseline knowledge^b^, n (%)**										.62
	Nothing	218 (49.3)		177 (51.2)		395 (50.1)				
	Some	217 (49.1)		166 (48)		383 (48.6)				
	A lot	7 (1.6)		3 (0.9)		10 (1.3)				
ICSEA^c^, median (IQR)			1100 (1032-1120)		1100 (1100-1120)		—^d^		.02	

^a^Pearson chi-square test for categorical variables, and the Kruskal-Wallis test for continuous variables.

^b^Students were asked to nominate which of these 3 statements was the most accurate: I don't know anything about meningococcal disease, I know a little about meningococcal disease, and I know a lot about meningococcal disease.

^c^ICSEA: Index of Community Socio-Educational Advantage.

^d^Not applicable.

Because they answered “I’m not sure/I don’t know” to all the questions on the survey, 33 participants in the control group (33/346, 9.5%) and 18 participants in the MIApp group (18/442, 4.1%) had a score of 0 for the pre-intervention survey. The median pre-intervention correct score in both the MIApp and control cohorts was 6/16 (MIApp: IQR 4-8; control: IQR 3-9), and the median postintervention correct score in both the MIApp and control cohorts was 14/16 (MIApp: IQR 12-15; control: IQR 13-15). The median improvement in score for the total cohort was 7/16 (IQR 5-10). The median improvements in score were 7/16 (IQR 5-9) for the MIApp arm and 8/16 (IQR 5-11) for the control arm (*P*<.001). Statistical equivalence in mean improvement between arms was demonstrated, with the confidence intervals for the mean difference falling within 2 points (MIApp vs control: mean difference=–1.036, lower limit=–1.569, upper limit=–0.503). Score improvement differed by year group, with students in years 9 and 10 having slightly lower median improvements in score (7 and 6, respectively) than those in years 7 and 8, who both had median improvements of 8 (*P*<.001). No statistically significant differences in median score improvement were noted by gender (*P*=.20; Table 3).

**Table 3:** Median number of correct scores (from a total score of 16) pre- and postintervention and changes in the median score by year and gender.

Characteristic		MIApp^a^ (n=442)				F2F^b^ (n=346)				Total sample (N=788)				*P* value^c^
		Pre-intervention, median (IQR)	Postintervention, median (IQR)	Change in the score, median (IQR)	Pre-intervention, median (IQR)		Postintervention, median (IQR)	Change in the score, median (IQR)	Pre-intervention, median (IQR)		Postintervention, median (IQR)	Change in the score, median (IQR)		
**Year**														<.001
	7	6 (3.5-8)	14 (12-15)	7 (5-9.5)	6 (3-8)		14 (13-15)	8 (6-11)	6 (3-8)		14 (13-15)	8 (5-10)		
	8	6 (3.75-8)	14 (13-15)	7 (4.75-10)	6 (2-8)		15 (14-15)	9 (6-12)	6 (3-8)		14 (13-15)	8 (5-10.25)		
	9	7 (4-8)	14 (13-15)	7 (5-10)	6 (4-9)		14 (13-15)	8 (5-11)	6 (4-8)		14 (13-15)	7 (5-10)		
	10	7 (6-9)	14 (12-15)	5 (4-8)	7 (5.75-9.25)		15 (14-15)	7 (4-9)	7 (6 9)		14 (13-15)	6 (4-8)		
**Gender**														.20
	Male	7 (4-9)	14 (12-15)	7 (4-9)	6 (3-8)		14 (13-15)	8 (5-11)	6 (3-8)		14 (13-15)	7 (5-10)		
	Female	4 (4-8)	14 (13-15)	7 (5-10)	6 (3-9)		14 (13-15)	8 (5-11)	6 (4-9)		14 (13-15)	8 (5-10)		
	Other	6 (5-8)	13 (8-14)	5 (2-8)	6 (2-7)		14 (13-15)	8 (6-11)	6 (4-7.75)		13 (11-14)	6.5 (2.5-9)		

^a^MIApp: Meningococcal Immunisation Awareness, Prevention and Protection app.

^b^F2F: face to face.

^c^Kruskal-Wallis test.

Table 4 shows the percentage of MIApp participants responding to questions about attitudes toward and engagement with the game; because these questions were not compulsory, not all students responded. Most participants responded positively: “I thought MIApp was easy to use” (348/426, 81.7%); “It was clear when I was playing what I was supposed to do next” (342/427, 80.1%); “MIApp was more enjoyable than a presentation” (326/426, 76.5%); “I felt very confident using MIApp” (315/423, 74.5%); and “I would like to use educational games like this more often” (313/424, 73.8%). Few found it difficult to use or needed help: “I needed to learn a lot more before I could understand MIApp” (58/426, 13.6%); “Finding things in MIApp was too hard” (45/428, 10.5%); “I found using MIApp a bit overwhelming” (37/424, 8.7%); and “I needed help from my teacher to be able to use MIApp” (27/427, 6.3%).

**Table 4:** Student attitudes about the Meningococcal Immunisation Awareness, Prevention and Protection app (MIApp) by year.

Statement	Agreement with the statement, n (%)				
	Year 7 (n=215)	Year 8 (n=60)	Year 9 (n=118)	Year 10 (n=49)	Total sample (n=442)
I would like to use educational games like this more often	162 (79.4)^a^	45 (76.3)^b^	73 (64)^c^	33 (70.2)^d^	313 (73.8)^e^
Finding things in MIApp was too hard	23 (11.1)^f^	3 (5.1)^b^	10 (8.8)^c^	9 (19.1)^d^	45 (10.5)^g^
It was clear when I was playing what I was supposed to do next	166 (80.2)^h^	48 (81.4)^b^	91 (79.8)^c^	37 (78.7)^d^	342 (80.1)^i^
I needed help from my teacher to be able to use MIApp	15 (7.2)^h^	2 (3.4)^b^	5 (4.4)^c^	5 (10.6)^d^	27 (6.3)^i^
MIApp was more enjoyable than a presentation	159 (76.8)^h^	46 (78)^b^	86 (75.4)^c^	35 (76.1)^j^	326 (76.5)^k^
I thought the features of on the screen on MIApp were confusing	27 (13.1)^l^	3 (5.1)^b^	7 (6.2)^m^	12 (25.5)^d^	49 (11.5)^n^
I thought MIApp was easy to use	168 (81.6)^l^	48 (81.4)^b^	92 (80.7)^c^	40 (85.1)^d^	348 (81.7)^k^
I found using MIApp a bit overwhelming	17 (8.3)^o^	3 (5.1)^b^	12 (10.6)^m^	5 (10.6)^d^	37 (8.7)^e^
I felt very confident using MIApp	151 (73.7)^o^	44 (74.6)^b^	85 (75.9)^p^	35 (74.5)^d^	315 (74.5)^q^
I needed to learn a lot more before I could understand MIApp	29 (14.1)^l^	9 (15.3)^b^	14 (12.3)^c^	6 (12.8)^d^	58 (13.6)^k^

^a^n=204.

^b^n=59.

^c^n=114.

^d^n=47.

^e^n=424.

^f^n=208.

^g^n=428.

^h^n=207.

^i^n=427.

^j^n=46.

^k^n=426.

^l^n=206.

^m^n=113.

^n^n=425.

^o^n=205.

^p^n=212.

^q^n=423.

A total of 255 participants from both groups completed nonduplicated (by unique identifier) responses for the 3-month follow-up survey, with a lower response rate for the MIApp arm (MIApp: 97/442, 22%; control: 158/346, 45.7%; *P*<.001). Of these, only 89 responses could be linked to the primary questionnaire results using the unique study identifier (MIApp: 50/442, 11.3%; control: 39/346, 11.3%; *P*=.99). The inability to match responses to the original survey for the majority of respondents precludes an analysis at follow-up by demographic factors.

The 3-month follow-up survey asked participants to answer the same set of questions related to the 9 key knowledge areas and indicate if they participated in the MIApp or control arm on the day of the trial. The median total correct scores at the 3-month follow-up were 11/16 (IQR 9-13) and 12/16 (IQR 9-13) for the MIApp and control arms, respectively (*P*=.86). A sensitivity analysis only including the 89 participants who could be matched to the trial surveys produced similar results (correct scores of 12/16 and 13/16 for the intervention and control arms, respectively).

Although there was a slight decline in knowledge at the 3-month mark, this again demonstrates that there was similar knowledge retention in both groups 3 months after the initial lesson. Overall, these results suggest very similar improvements in the pre-post intervention scores by intervention group, year, and gender. This suggests that students either receiving an educational session via an AYF presenter or playing the MIApp can expect to improve their knowledge of IMD in the 9 key areas outlined in the previous sections.

## Discussion

### Principal Findings

This study demonstrated the efficacy of a serious game, MIApp, in improving knowledge about IMD in high school–aged children at equivalent levels to that of a face-to-face presentation delivered by a content expert. Serious games are attracting significant attention for promoting health education among children and adolescents [4-6,45], yet there is still a need to understand their effectiveness [22,27,30]. Our literature review suggested that, although serious games may offer a viable alternative to traditional pedagogical methods in terms of knowledge improvement, behavioral and attitudinal change, and engagement, the varied nature of serious games used in health promotion and the lack of standardized tools to measure outcomes mean that the results are heterogeneous, often with small sample sizes and high attrition [18].

This study compared the efficacy of the serious game MIApp with a traditional classroom health education model on knowledge about IMD in a sample of secondary school students. The results demonstrated that this serious game (1) significantly improved knowledge about the disease among adolescents, (2) had an outcome statistically equivalent to that of the current face-to-face educational approach, and (3) was easy to use and more enjoyable. Importantly, the study showed that gains in knowledge from playing the serious game MIApp were sustained 3 months later at equivalent levels to those in the control arm.

In line with recent research, this study supports the notion that age-appropriate, self-navigating serious games may be considered a positive learning tool for delivering school-based health education [25,26,46,47]. One fundamental feature of serious games is that they are “fun” and pedagogically sound [7-9]. As illustrated by the MIApp study, adolescents find digital game–based learning more enjoyable than a traditional classroom presentation, characteristics that are acknowledged as essential for effective engagement [48]. By providing a captivating learning environment, serious games can intrinsically and extrinsically motivate adolescents to promote active learning to understand health issues better [17,30,49]. Research also suggests that, through the provision of interactive engagement and immediate feedback, serious games can maintain students’ involvement for a longer period [50-52], supporting higher-level thinking and increasing learner independence [10].

With increasing numbers of children and adolescents in Australia using digital games [1], together with the immediate transition to digital learning during the COVID-19 pandemic [53], serious games offer a unique educational platform to improve the reach of health education [5,28,54,55]. As a self-guided resource not limited to staff facilitating an education session, serious games can support teachers to provide health education around topics they have limited knowledge about or may find difficult to discuss [7]. Moreover, this study revealed that students using MIApp also retained their IMD knowledge over 3 months, as did those who attended a face-to-face educational presentation. Although research has suggested serious games might be more beneficial for long-term retention of health information due to their enjoyable format [4], to date, most studies have focused primarily on short-term pre- and posttests [25].

Although this study was conducted with schools in primarily medium-to-high ICSEA areas of Perth, WA, as shown in Table 2, its relevance needs to be assessed in rural settings where cases of IMD are higher; the preliminary findings of this study suggest MIApp could represent an emerging resource in the space of meningococcal education for adolescents and young adults in Australia. Key features underlying MIApp are known to promote motivational learning, improve knowledge, and promote engagement, which can increase educational achievement [56,57], and are likely responsible for the positive findings observed in our study [46,52,58]. MIApp is freely downloadable from the App store [59] on personal devices or is available on the AYF website [60] as a desktop version, accessible to all, including those regional and rural communities that previously would not have had the resources or access to receive the face-to-face guest presentations. Findings of this study can help teachers to feel more assured that students are gaining the same learning opportunity through an interactive and engaging app as they would receive from a qualified presenter.

As shown in previous research [26,31,47,56], engaging key stakeholders in the co-design, development, testing, and refining of the serious game MIApp was essential; adopting a co-design approach with all stakeholders is useful in promoting health communication interventions and enhancing the acceptability, usability, and utility of the serious game [26]. For MIApp, collaborators included an interdisciplinary team of researchers, experts, and community partners with varied expertise in education, health and computer science, and game development. This collaboration facilitated important development and design improvements of the game interface, mechanism, health content, and pedagogy for students and ensured that the social and cultural interests of the targeted adolescent group were included.

For greater influence, serious games for health education and promotion should be designed in a way that enables them to be embedded in national curriculum, as this gives teachers the opportunity to combine gameplay with the mandated curriculum to address learning outcomes [15,16,18]. MIApp was aligned specifically with the teaching and learning HPE curriculum as outlined by the Australian curriculum and, more specifically, the lower secondary year-level syllabi for the HPE Learning Area in WA [40]. Additionally, a free downloadable teaching and learning curriculum companion of activities was developed for teachers to assist them to cognitively build and support student understanding of concepts and health-enhancing skills and dispositions related to meningococcal awareness and prevention and is available from the AYF website. These learning activities have also been aligned to meet the teaching and learning requirements of the HPE curriculum.

The overall educational package draws on 5 interrelated ideas underpinning the pedagogy in the delivery of HPE in Australia. The broader dissemination of this tool by teachers as a public health intervention could support the digital implementation of national efforts to reduce IMD among adolescents. The demonstrated efficacy of MIApp means that educators, particularly in rural and remote areas without previous access to specialist education sessions about IMD, can deploy this game in the classroom to provide their students with self-guided education supported by teaching resources.

### Limitations

There were several limitations to this research. First, a major limitation to this study was the low response rate and match rate for the 3-month follow-up survey. There was a relatively low rate of complete responses (255/788, 32.4%) for the 3-month follow-up; the response rate was significantly lower for the MIApp group (97/442, 21.9%) than the control group (158/346, 45.7%). Attrition for this follow-up survey was expected to be low, and our literature review highlighted significant issues with attrition experienced by researchers running similar interventions [18]. Although attrition was built into the sample size calculation, the 3-month follow-up response rate was still much lower than anticipated. Future projects may consider methods to improve response rates, including incentives for completing follow-up surveys.

Further, only 35% of completed surveys could be linked to the identifiers for the pre-postintervention arm, due to students entering an identifier that did not match the one created on the day of trial. The method for creating a unique identifier involved using parts of key demographic information (name and date of birth) that were considered robust and unlikely to change over the study period. Despite this assumption, 65% of identifiers and school codes did not match those created on the day of the trial. A more robust identifier may be considered for future trials where follow-up data need to be linked to data collected on the day of the trial.

Finally, a significantly lower proportion of responses in the 3-month follow-up was received from those in the intervention arm than those in the control arm. The implications of this difference are that, potentially, respondents from the intervention arm were systematically different from those who did not respond. If respondents reflect those who were more likely to retain information, this may have biased the results. The overall low response rate in both groups may suggest this bias would be present in both; however, residual bias from the difference in response rates between groups may still be present. A sensitivity analysis using only surveys that were able to be linked to the primary trial data, which also allows independent confirmation that the correct trial arm was selected on the follow-up survey, yielded a response rate that was *not* significantly different between groups. In addition, the data from these groups confirmed the 1-point difference in correct responses between the intervention and control arms reported in the larger follow-up cohort, which was within the predetermined equivalence threshold. This increases our confidence that the results reported in the larger follow-up sample were reflective of the larger cohort.

Despite these limitations, the completed surveys suggested that many key messages delivered via either MIApp or in a face-to-face presentation are retained 3 months after a single 30-minute session. Future studies assessing the longer-term efficacy of serious games for health promotion may consider outcome measures of behavior change that do not rely on participant response rate (eg, vaccination and infection rates).

The overall findings of this study suggest that serious games, such as MIApp, represent an effective strategic avenue for the use of digital technology to engage adolescents in learning about key health issues. The research found both delivery methods to be similar in improving knowledge and attitudes toward meningococcal carriage and disease signs, symptoms, and prevention. Coupled with greater reported enjoyment, if well-designed, serious games could be used in national health education. The strengths of this study included the engagement of a range of key stakeholders in its planning and design to improve outcomes and ensure specificity of knowledge relating to the curriculum and possibilities for content inclusion in the teaching and learning of HPE in Australia. Although further investigations are needed to explore any minor changes required for the delivery in the diverse socioeconomic, cultural, and geographic settings of Australia, this study has illustrated the efficacy of the MIApp game. Moreover, data generated in this study can support evidence for the development of future serious games for adolescent health education worldwide. Today, in our digital landscape, greater understanding of the game learning interests of adolescent students is paramount to actively promote their active engagement in health education.

## Appendix

Multimedia Appendix 1 MiApp Post Study Questionnaire.

Multimedia Appendix 2 Screenshots from MiApp game.

Multimedia Appendix 3 Screenshots #2 from MiApp game.

Multimedia Appendix 4 Screenshot #1 from MiApp game.

Multimedia Appendix 5 Amanda Young Foundation classroom presentation.

Multimedia Appendix 6 MIApp Extended User Guide.

Multimedia Appendix 7 CONSORT-EHEALTH (Consolidated Standards of Reporting Trials of Electronic and Mobile Health Applications and Online Telehealth) form for the Meningococcal Immunisation Awareness, Prevention and Protection app (MIApp) study.

## Abbreviations

AYF: Amanda Young Foundation
ECU: Edith Cowan University
HPE: Healthy and Physical Education
ICSEA: Index of Community Socio-Educational Advantage
IMD: invasive meningococcal disease
MIApp: Meningococcal Immunisation Awareness, Prevention and Protection app
WA: Western Australia

## Data Availability

The data sets generated during and/or analyzed during the current study are available from the corresponding author on reasonable request.
