# Supplementary figures and images for: Evaluating the Efficacy of a Serious Game to Deliver Health Education About Invasive Meningococcal Disease: Clustered Randomized Controlled Equivalence Trial
Source: JMIR Serious Games. 2025 Feb 11;13:e60755. doi: 10.2196/60755 (PMC11862768; doi:10.2196/60755)

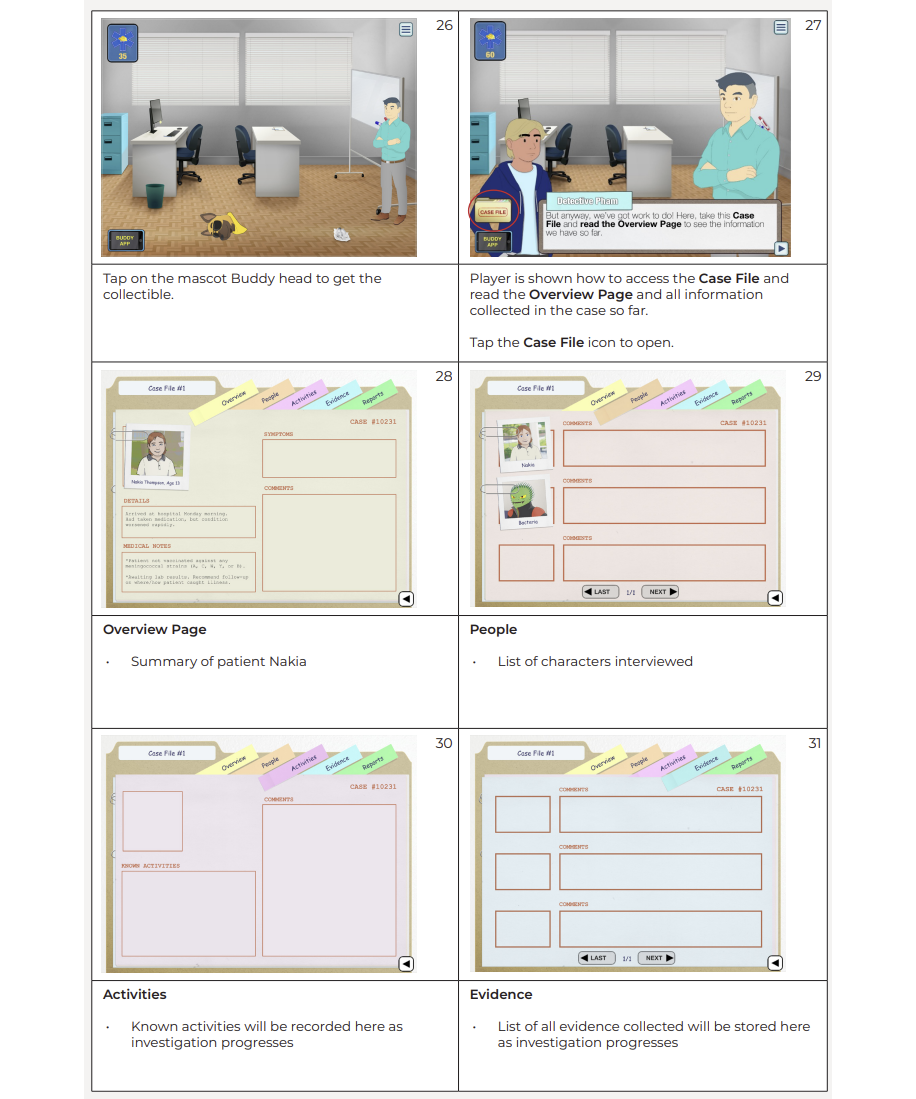

Supplement: Multimedia Appendix 2 [file games_v13i1e60755_app2.png]

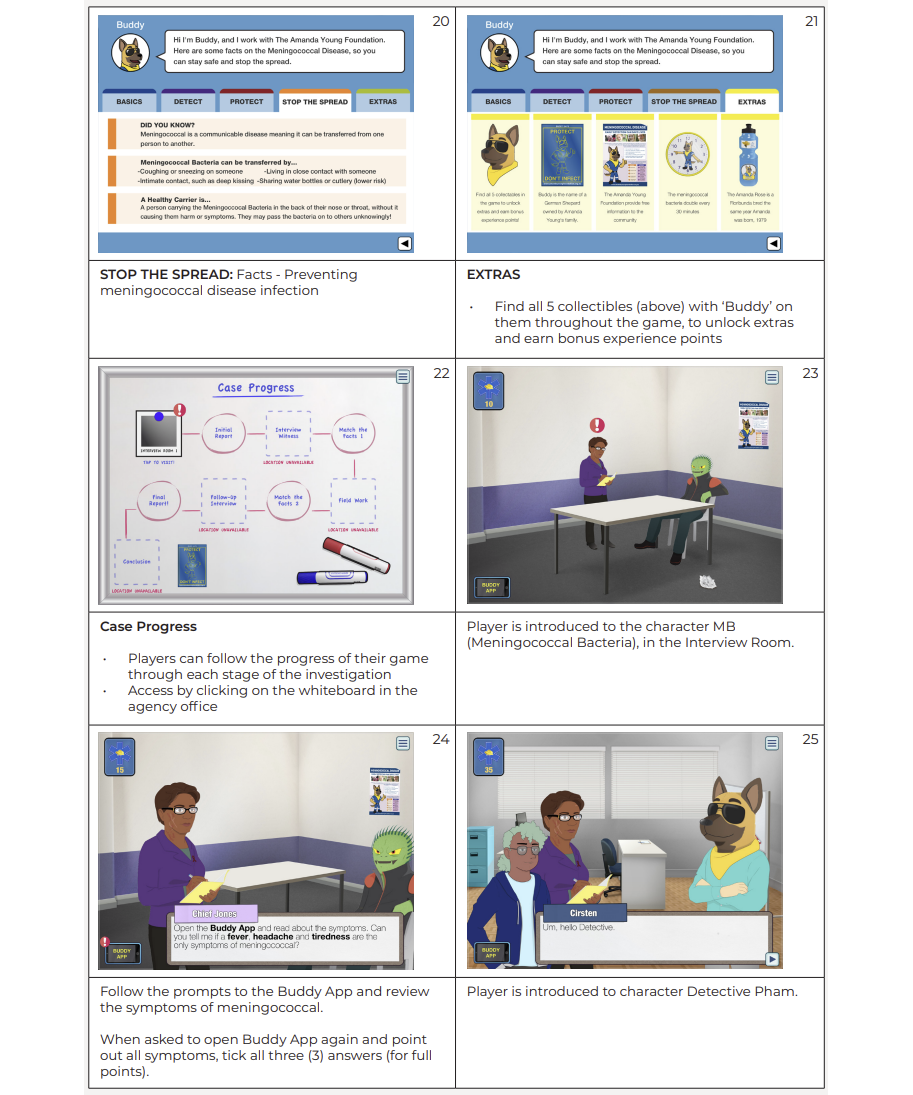

Supplement: Multimedia Appendix 3 [file games_v13i1e60755_app3.png]

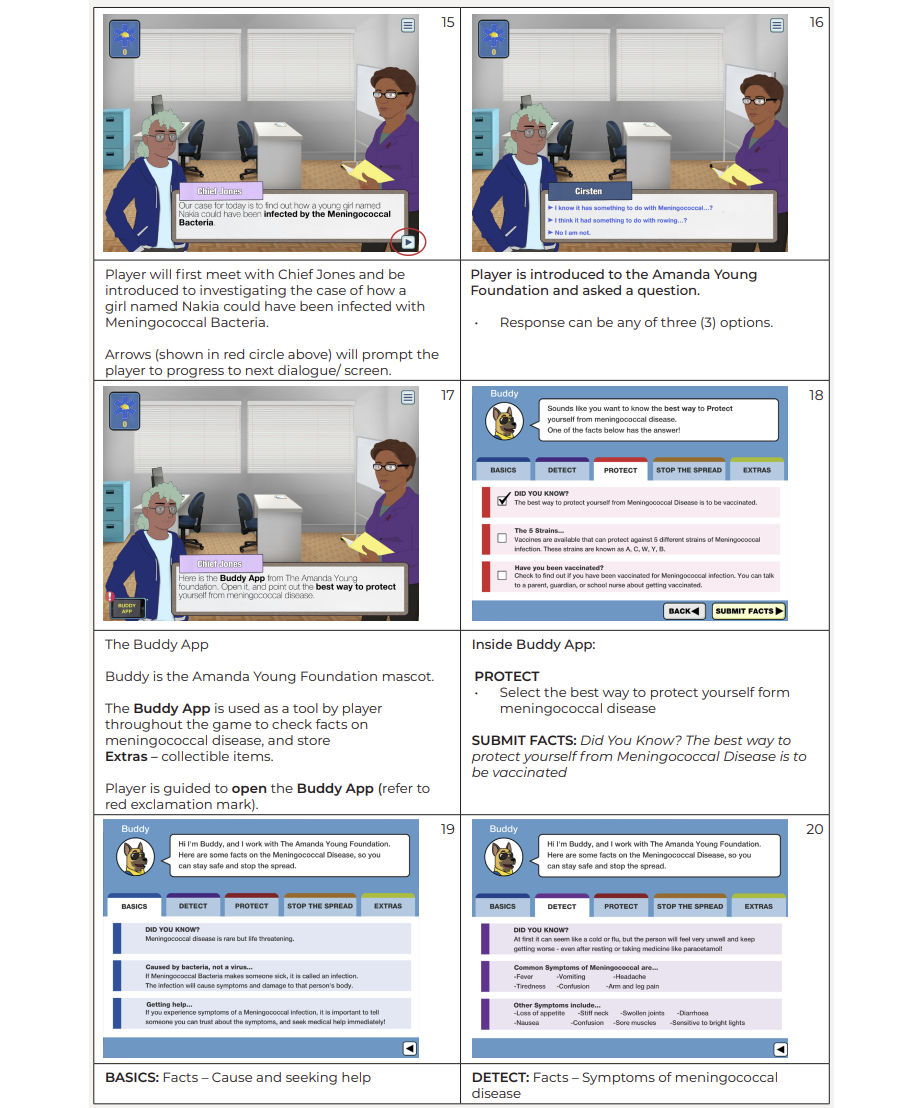

Supplement: Multimedia Appendix 4 [file games_v13i1e60755_app4.png]
