# Supplementary material for: Evaluating the Efficacy of a Serious Game to Deliver Health Education About Invasive Meningococcal Disease: Clustered Randomized Controlled Equivalence Trial
Source: JMIR Serious Games. 2025 Feb 11;13:e60755. doi: 10.2196/60755 (PMC11862768; doi:10.2196/60755)
Supplement: Multimedia Appendix 5 [file games_v13i1e60755_app5.pdf]

The Amanda Young Foundation

fighting meningococcal disease

AWARENESS PREVENTION & PROTECTION

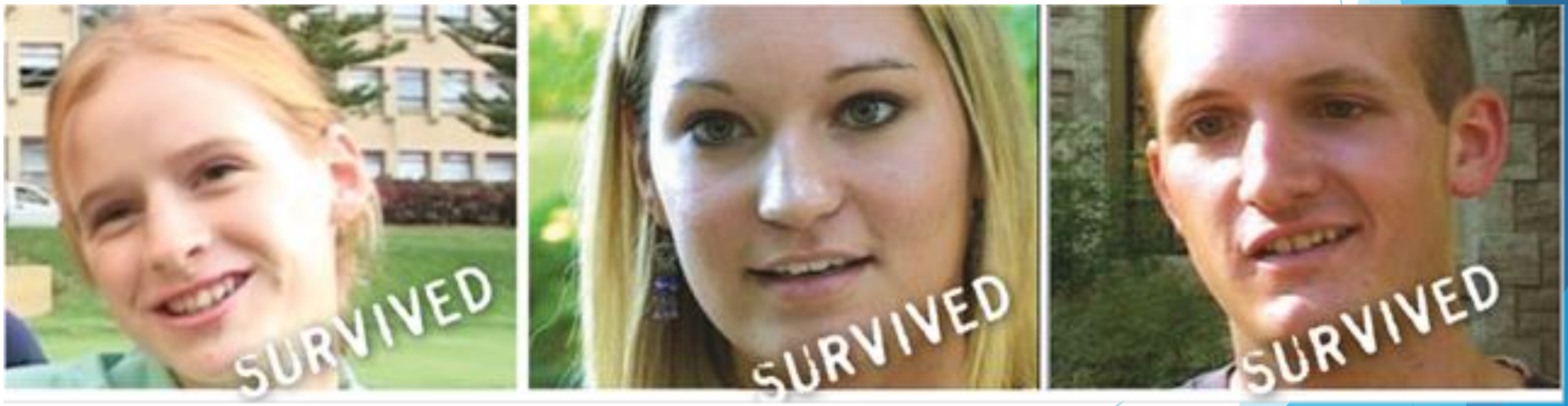

The
Amanda Young
Foundation
fighting meningococcal disease

About Amanda

Amanda was a high achieving sportswoman and university student.

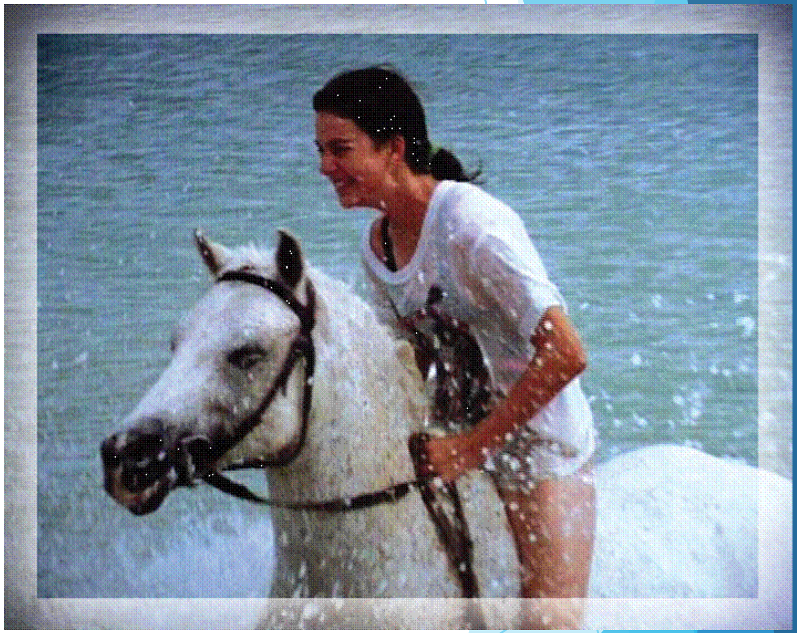

Amanda contracted **meningococcal septicaemia** at a rowing regatta in Sydney in 1997. She died, aged 18, less than 24 hours after the first sign of symptoms.

What you will learn today.

- **Awareness**
- **Prevention**
- **Protection**

So that you may remain healthy-
protect yourself and others.

The
Amanda Young
Foundation
fighting meningococcal disease

Awareness……..

What is Meningococcal Disease?

- acute infection caused by **bacteria** not a virus
- different strains of the disease - strains C, B, W & Y found in Australia
- meningococcal bacteria can double every 30 minutes
- early detection and medical treatment vital to survival

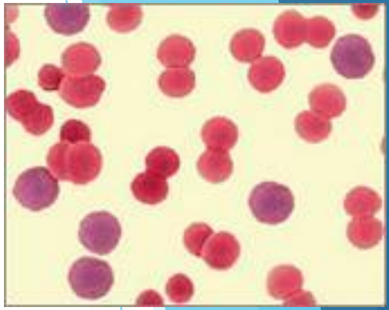

The Amanda Young Foundation

fighting meningococcal disease

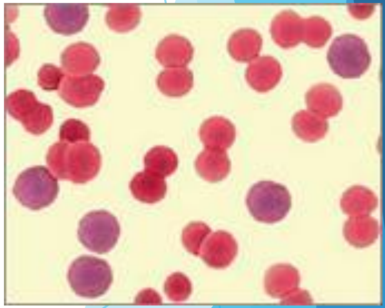

Awareness……..

What is Meningococcal Disease continued…

- Teenagers and babies are the most at risk groups
- The disease progresses rapidly with patients at risk of death within hours
- Early symptoms resemble common, relatively harmless illnesses like cold or ‘flu meaning early diagnosis can be difficult

The Amanda Young Foundation
fighting meningococcal disease

VIDEO

The
Amanda Young
Foundation
fighting meningococcal disease

DON’T CATCH THE KILLER…..

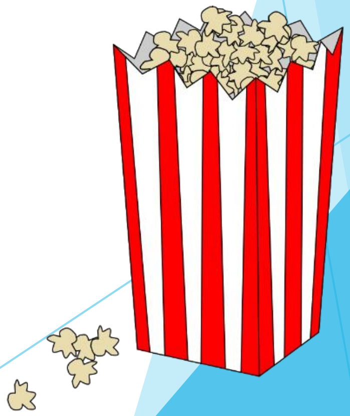

AMANDA YOUNG FOUNDATION

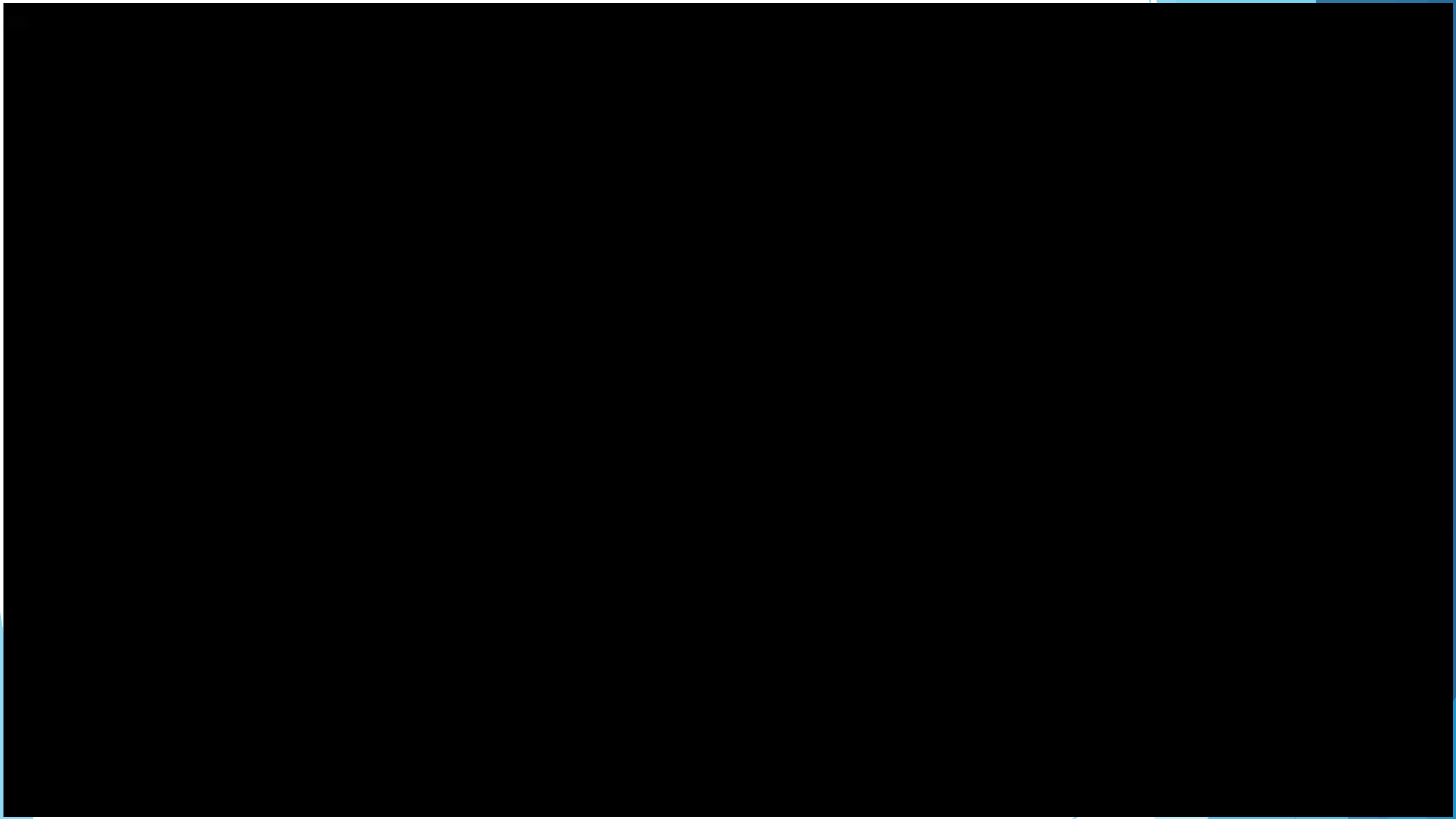

DON’T CATCH THE KILLER – Part 1

Awareness……..

National Graph of MD Cases since 2008

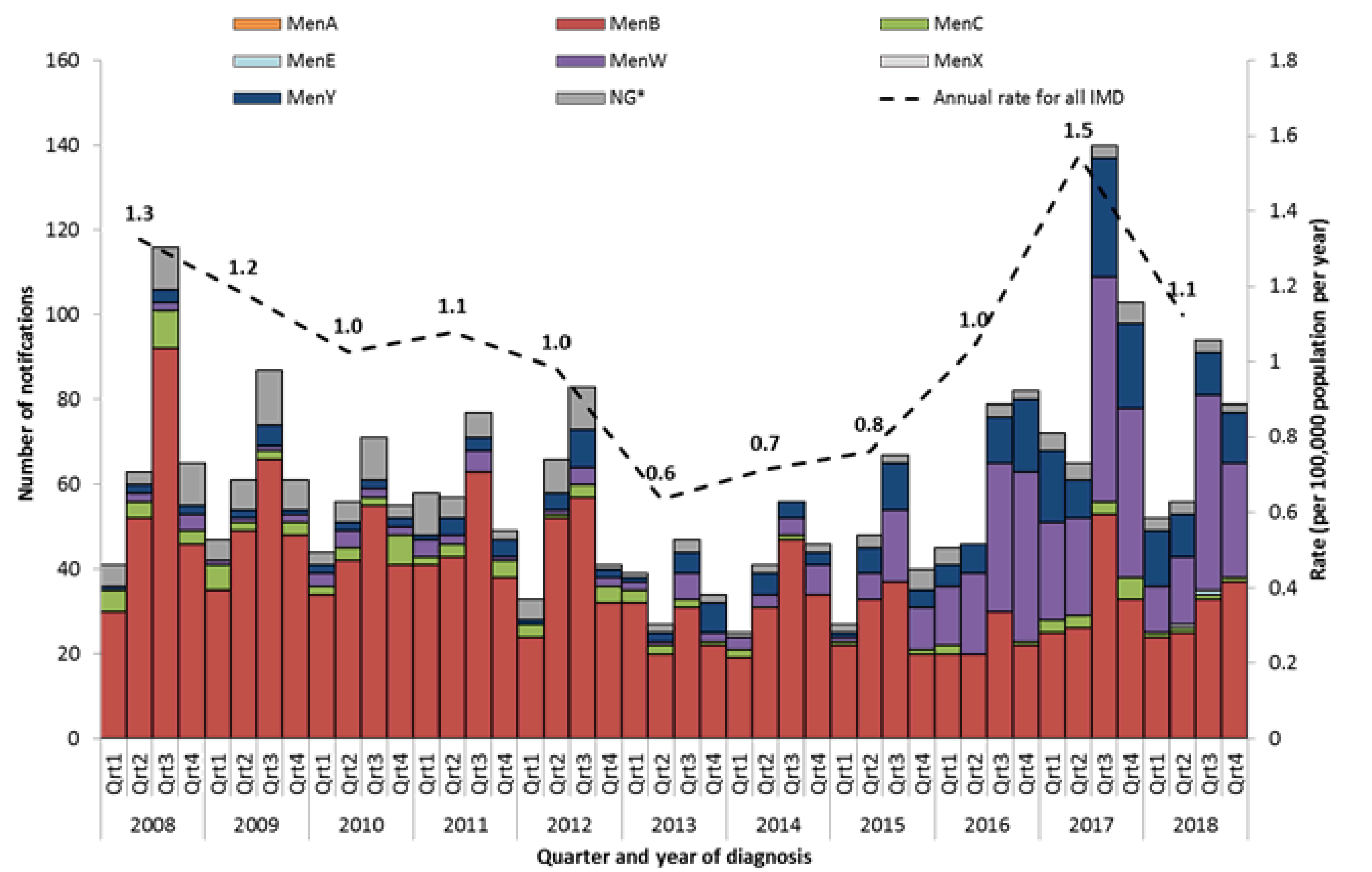

Source – www.health.gov.au

Awareness……..

How is the Disease Spread?

- Meningococcal bacteria live naturally in the throat and back of nose.
- About 10-20% of people will be carrying the bacteria at any one time without ever becoming ill – they are called "healthy carriers".
- Bacteria spread through mucus and respiratory droplets from the throat: sneezing, coughing, kissing or perhaps sharing food or drinks.

The Amanda Young Foundation
fighting meningococcal disease

Awareness……..

Risk Factors

Reduced immunity due to recent illness or being rundown.

Multiple “intimate kissing” partners.

Smoking or exposure to smokers – smoking damages the lining of the mouth, nose & throat making it easier for the bacteria to invade.

Not being immunised

Sharing anything that has been in your mouth

The Amanda Young Foundation

fighting meningococcal disease

Awareness……..

How Common is Meningococcal Disease?

Classified as a rare disease in Australia

70% of patients make a full recovery

20% will have permanent disabilities, such as:

- Sight and hearing problems
- Liver and kidney failure
- Amputations – loss of fingers, toes or limbs

10% will die

The Amanda Young Foundation

fighting meningococcal disease

Types of Meningococcal Disease

Septicaemia: Infection of the blood

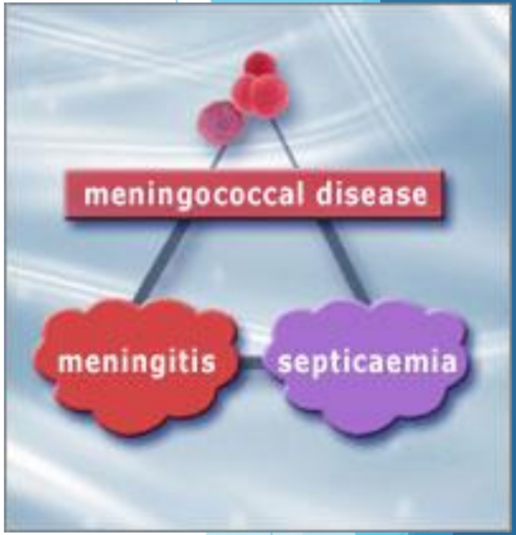

Meningitis: Infection of the membranes surrounding the spinal cord and brain (the "meninges")

Patients can have just septicaemia, just meningitis or both.

The Amanda Young Foundation
fighting meningococcal disease

QUIZ

The
Amanda Young
Foundation
fighting meningococcal disease

Have you been paying attention?

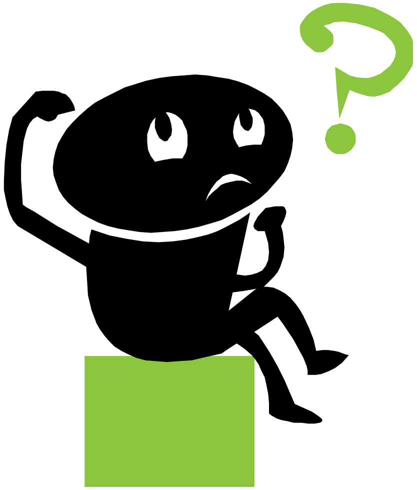

The Amanda Young Foundation

fighting meningococcal disease

Question 1

What is meningococcal disease?

a) An acute viral infection

b) An acute bacterial infection

Question 2

Meningococcal Disease is a common disease in Australia.

a. True
b. False

The Amanda Young Foundation

fighting meningococcal disease

Question 3

The meningococcal bacteria double

a) Every 60 minutes
b) Every 30 minutes

The Amanda Young Foundation

fighting meningococcal disease

Question 4

What percentage of the population are health carriers?

a. 10 to 20 percent

b. 30 percent

The Amanda Young Foundation

fighting meningococcal disease

Question 5

Meningococcal **meningitis** is…

a. Infection of the blood

b. Infection of membranes surrounding spinal cord and brain

The Amanda Young Foundation

fighting meningococcal disease

Question 6

Meningococcal Septicaemia ……

a. Bacteria enter the bloodstream and multiply uncontrollably, damaging the walls of the blood vessels and causing bleeding into the skin

b. Inflammation of the lining of the brain and other organs caused meningococcal bacteria

The Amanda Young Foundation

fighting meningococcal disease

Awareness……..

Meningococcal Disease Symptoms

**Meningococcal Septicaemia**	**Meningococcal Meningitis**
Shivering, chills, cold hands or feet, skin colour change	Severe Headache
Sudden, severe pain in arms, legs, joints or stomach	Stiff or painful neck
Fever that doesn’t respond to panadol, nausea/vomiting, maybe diarrhoea	Sensitivity to light
Drowsiness, loss of consciousness, rapid breathing	Drowsiness, loss of consciousness, fits
Spots or pinprick rash (develops to purple blotches)	A rash may develop in later stages

The Amanda Young Foundation

fighting meningococcal disease

The Septicaemic Rash

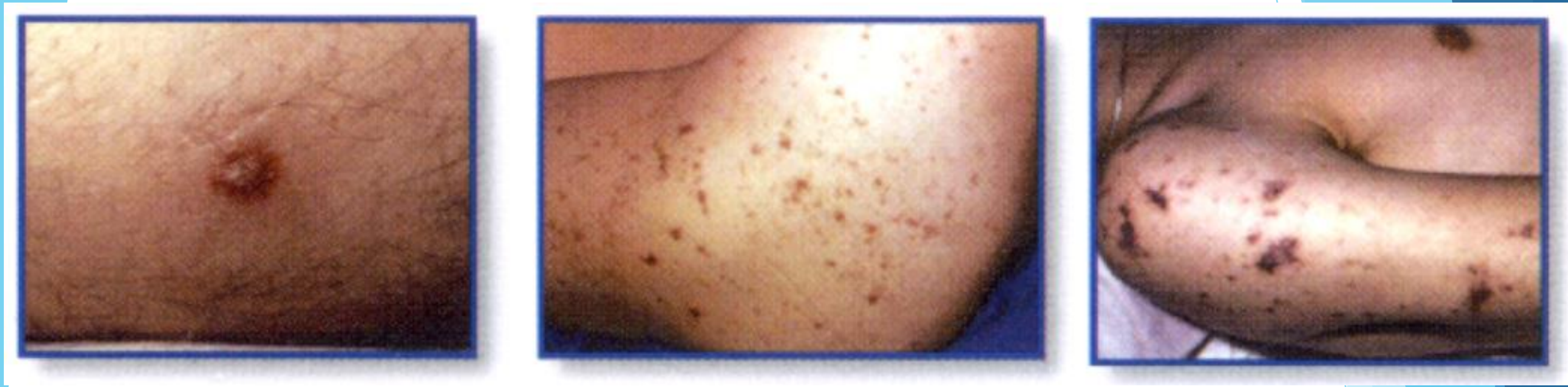

The rash appears in the final stages of septicaemia and is caused by blood leaking from damaged vessels into the skin.

IF A RASH APPEARS WITH SOME OF THE ABOVE SYMPTOMS

TREAT IT AS A MEDICAL EMERGENCY!

The
Amanda Young
Foundation
fighting meningococcal disease

Prevention &
Protection

Vaccination

- Vaccine for C-Strain (introduced in 2003) - part of routine vaccinations at 12 months. Very effective.

- Men B vaccine available from 2014 – see your GP (cost)

- ACWY strain

  - Free ACWY vaccine initiative for year 10 W.A. teenagers and all 15 to 19 year olds.

- Discuss with your parents if you are younger

Check out www.healthywa.wa.gov.au talk to your parents or school nurse if you are worried about side effects. The vaccine cannot give you meningococcal disease and is safe and effective.

The Amanda Young Foundation
fighting meningococcal disease

Prevention &
Protection

Prevention of Meningococcal Disease

Avoid sharing:

- Food and drinks
- Utensils
- Toothbrushes
- Cigarettes
- Mouthguards
- Anything that you put in your mouth.

The Amanda Young Foundation
fighting meningococcal disease

Stay away from crowds and public places when you are unwell.

The Amanda Young Foundation
fighting meningococcal disease

What to Do if You Suspect Meningococcal Disease

- If you are feeling extremely unwell, tell somebody you trust and ensure you both keep a close eye on your symptoms.
- Trust your instincts – you know how you "usually" feel when you're sick and if you have Meningococcal Disease you will feel far worse, very quickly.
- If you suspect meningococcal disease, ask an adult you trust to take you immediately to the doctor or hospital.
- If infected, treatment with intravenous antibiotics in hospital is required and those around you may be treated with oral antibiotics as a precaution.

Don’t Catch The Killer – Part 2

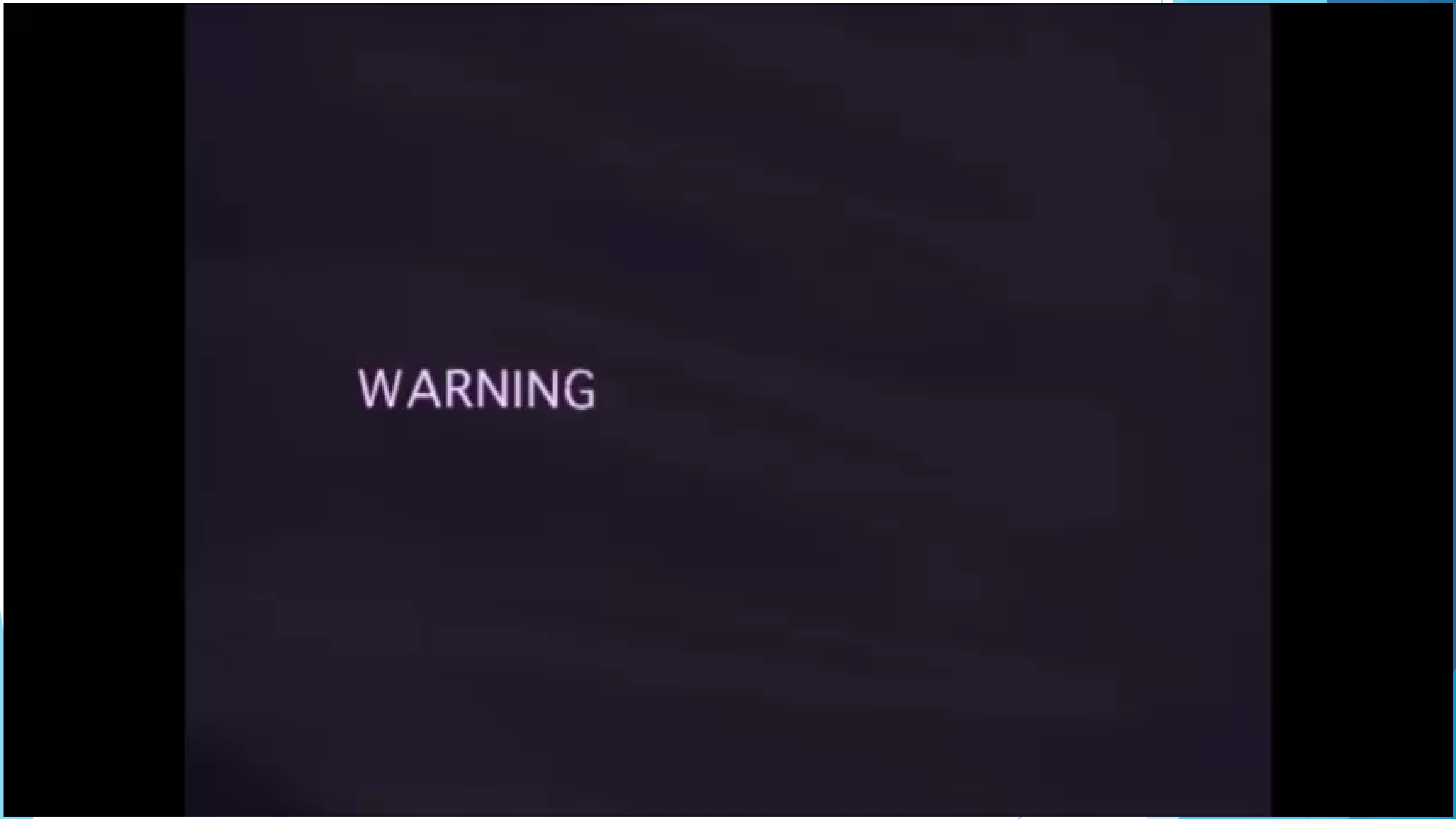

In Summary…

- Knowledge is power – share this information.
- Avoid transmission of saliva /mucus within reason. Stay in bed and away from crowds and exposure to coughs and sneezes when you are unwell.
- Tell somebody you trust when you are feeling unwell.
- If a rash appears with some meningococcal symptoms, treat it as a medical emergency!
- Talk to your doctor and/or school health nurse about vaccination.

#vaxandrelax

#vaccinatedontprocrastinate

The Amanda Young Foundation

fighting meningococcal disease

Sharing information…………

- turn to the person next to you
- share 3 important pieces of information you have learned today.

LIKE our FB page!

The
Amanda Young
Foundation
fighting meningococcal disease

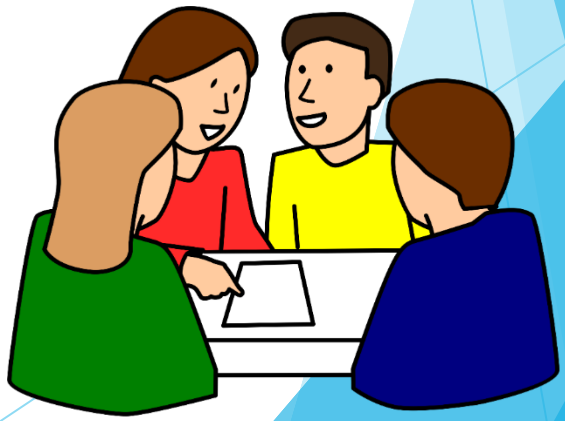

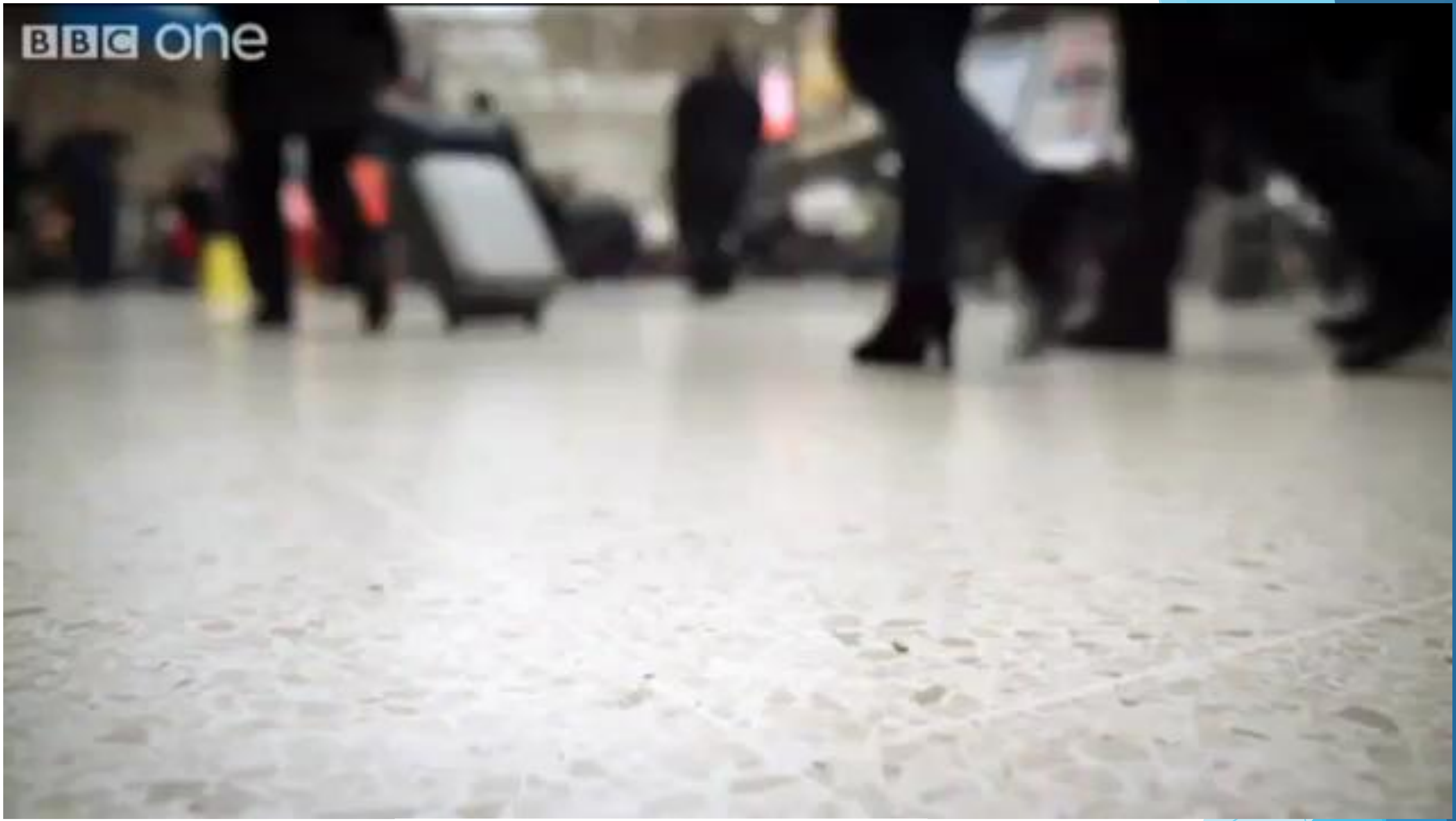

The
Amanda Young
Foundation

fighting meningococcal disease
