# Supplementary material for: Evaluating the Efficacy of a Serious Game to Deliver Health Education About Invasive Meningococcal Disease: Clustered Randomized Controlled Equivalence Trial
Source: JMIR Serious Games. 2025 Feb 11;13:e60755. doi: 10.2196/60755 (PMC11862768; doi:10.2196/60755)
Supplement: Multimedia Appendix 6 [file games_v13i1e60755_app6.pdf]

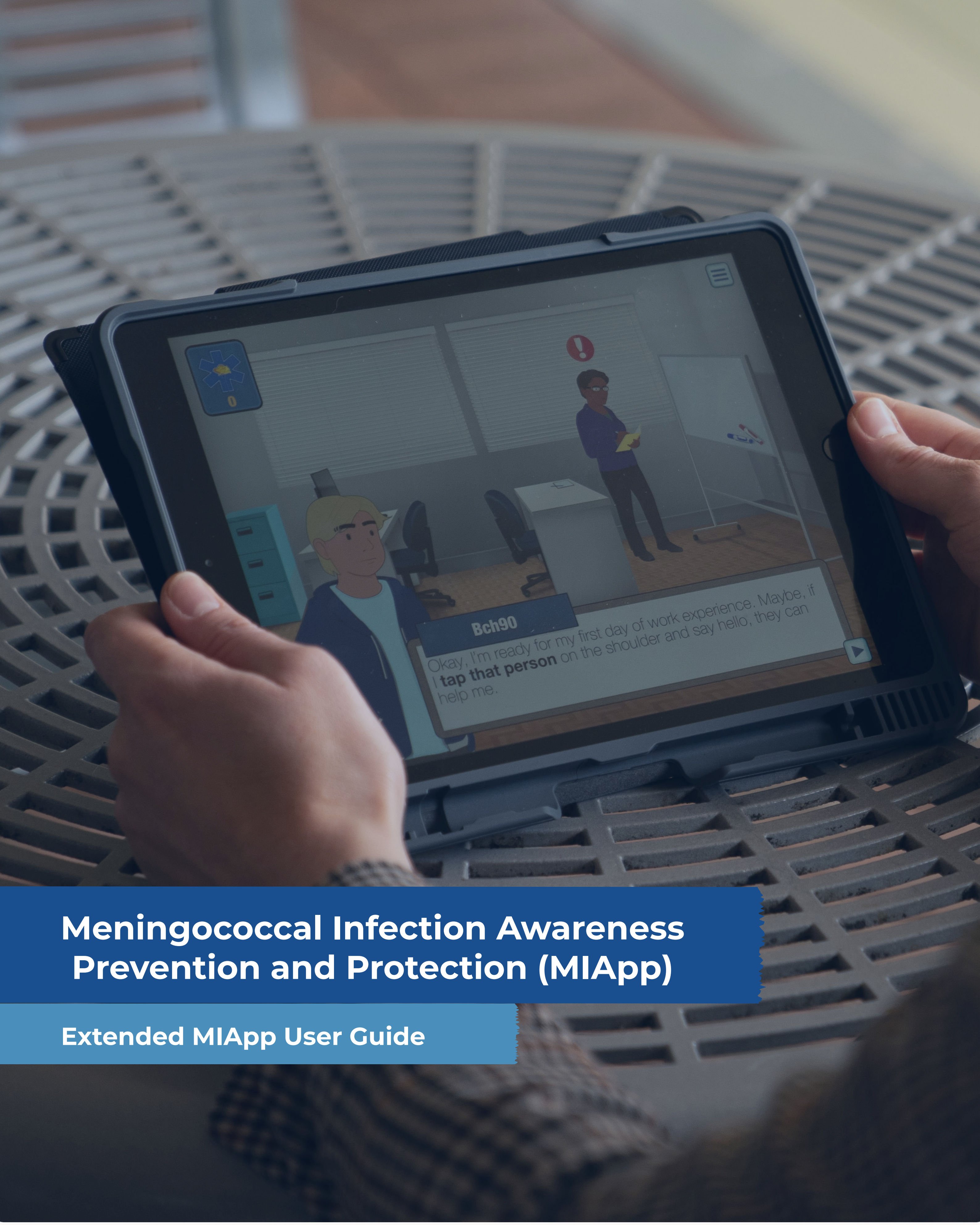


# Meningococcal Infection Awareness Prevention and Protection (MIApp)

## Extended MIApp User Guide

The Amanda Young Foundation

lotterywest

Government of Western Australia
Department of Health

Australia ECU Edith Cowan University

# Download Instructions

## MIApp for iPad – Apple App Store

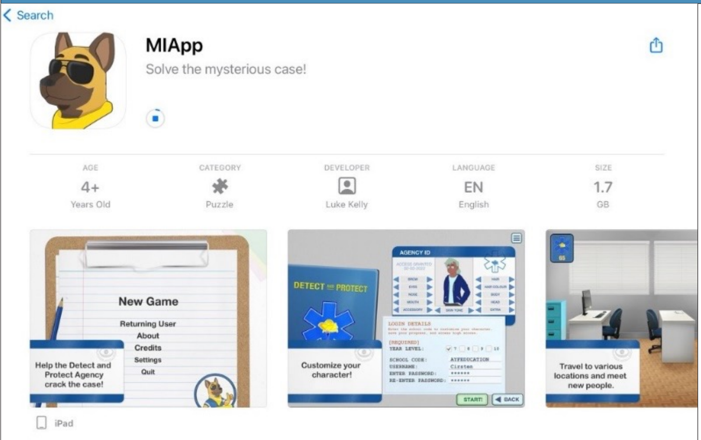


1. Open App Store app on your Apple iPad device
2. Available at: Search for app in search bar by typing 'MIApp' (free to download)
3. Click the Get button.
4. Download MIApp game for iPad from the [Apple App Store]

Note for educators: please allow up to 15 minutes for MIApp to download from the [App Store]

## MIApp for Windows PC

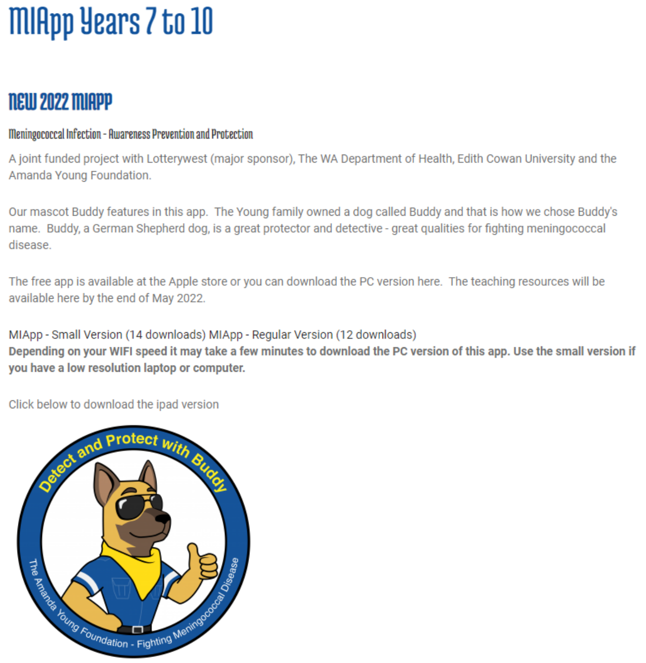

MIApp Years 7 to 10

NEW 2022 MIAPP

Meningococcal Infection - Awareness Prevention and Protection

A joint funded project with Lotterywest (major sponsor), The WA Department of Health, Edith Cowan University and the Amanda Young Foundation.

Our mascot Buddy features in this app. The Young family owned a dog called Buddy and that is how we chose Buddy's name. Buddy, a German Shepherd dog, is a great protector and detective - great qualities for fighting meningococcal disease.

The free app is available at the Apple store or you can download the PC version here. The teaching resources will be available here by the end of May 2022.

MIApp - Small Version (14 downloads) MIApp - Regular Version (12 downloads)

**Depending on your WIFI speed it may take a few minutes to download the PC version of this app. Use the small version if you have a low resolution laptop or computer.**

Click below to download the ipad version



1. In web browser, visit the [Amanda Young Foundation website]
2. Download the zip file
   - Regular build
   - Small (optimised for smaller screens)
3. Extract the file
4. Run the .exe application within the folder

## Additional information for Windows PC build

The windows build forces an aspect ratio of 4:3 with a resolution of 1024×768 (as the game was originally designed and built for an iPad). Due to the 4:3 aspect ratio, the game will launch in a window and will not take up the whole width of a standard monitor. Achieving this would distort the graphics to the detriment of the user experience.

The PC build was tested on various monitor resolutions, including:

- 3840 x 2160
- 1920 x 1080

The windows build has been tested on various monitor sizes from 21", 24", 27" and on a 74" Windows-based touch table. MIApp for Windows works across these commonly found monitor sizes (laptops and desktop computers).

# MIApp Player Guide

1

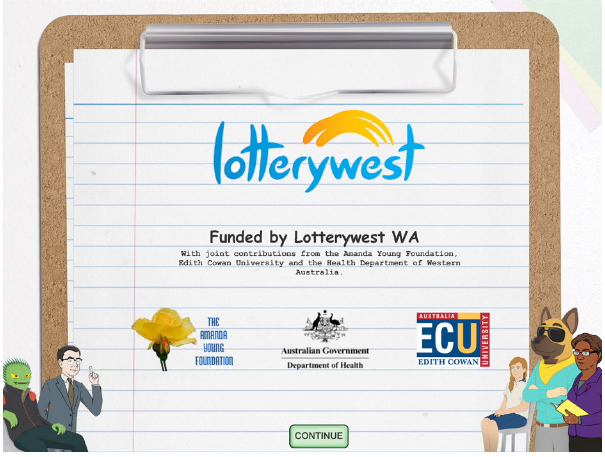


First Screen – Acknowledgements

- Player select Continue

2

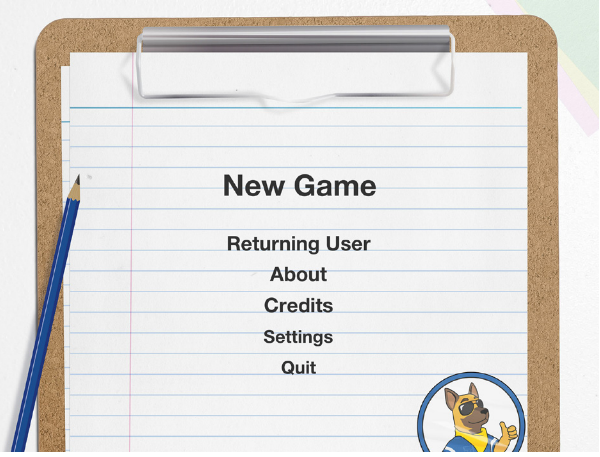


Home screen

Players can choose either

- New Game
- Returning User
- About
- Credits
- Settings
- Quit

3

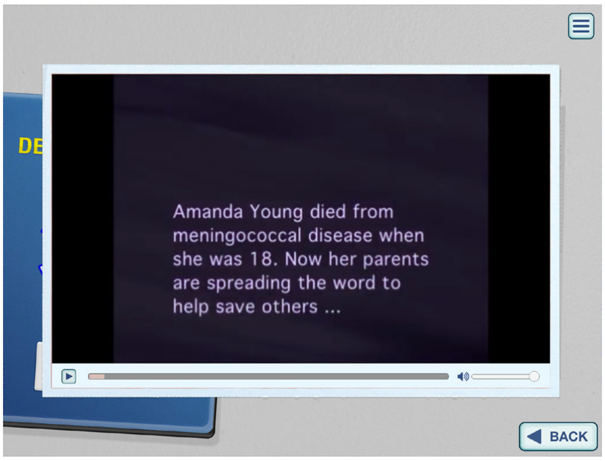


About – Amanda Young Foundation background information

4

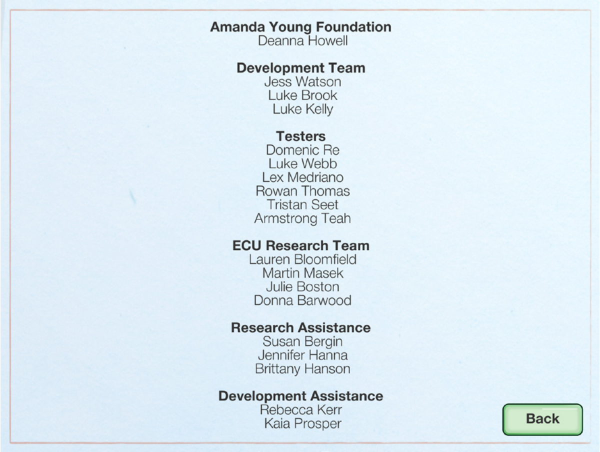


Credits

5

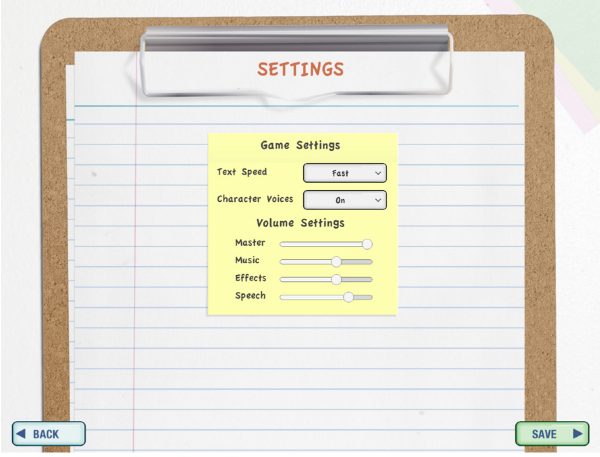


Settings

- Game settings can be adjusted for preference of text speed, character voices ON (default) or OFF, and volume settings

6

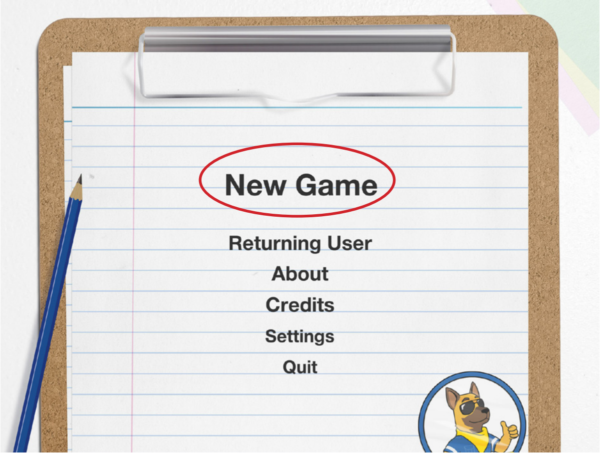


New Game

- Players select New Game

7

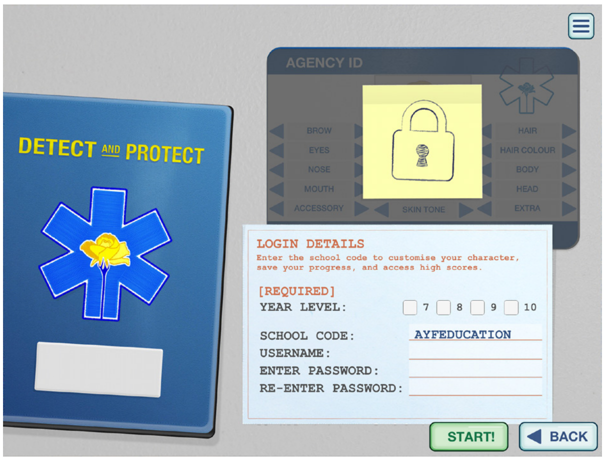


Login Details

Player to enter login details:

- Player to enter **Year Level**
- School code: **AYFEDUCATION**
- Username – 3 or more characters
- Password – 5 or more characters

8

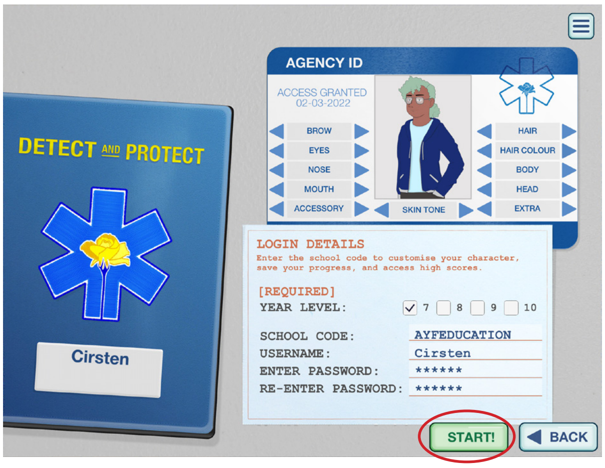


Player can then customise an avatar in the **Agency ID** card

- Using the arrows to change hair, eyes, accessories etc.
- Then select **START!** to commence game

9

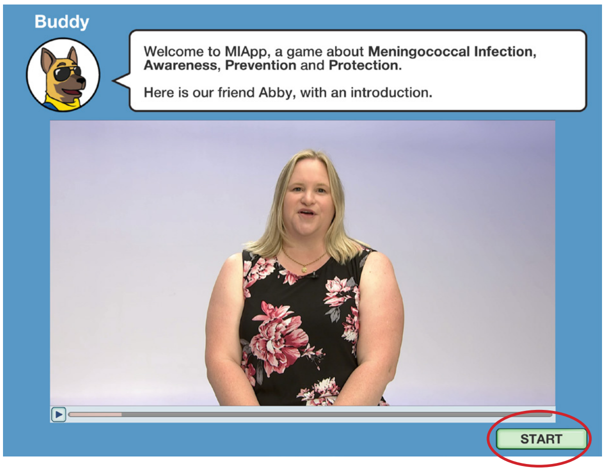


Introduction video – Abby, The Amanda Young Foundation

- Player to watch short introduction video
- Then select START to commence game

10

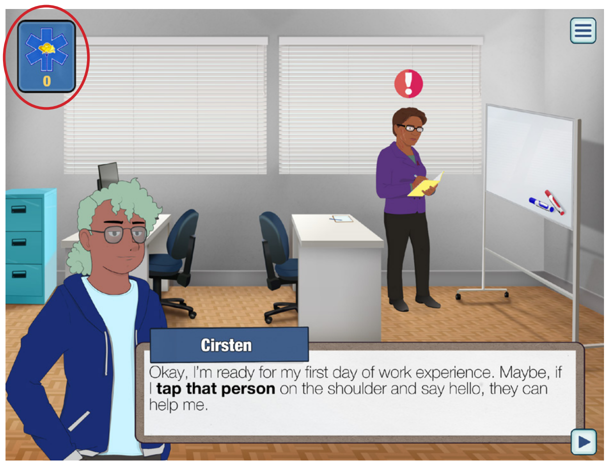


Player will first enter the agency office for their characters' first day of work experience at the bacteria tracking agency, **Detect and Protect**.

**Note:** Throughout game play, the exclamation mark will prompt the player to click on that character or item

11

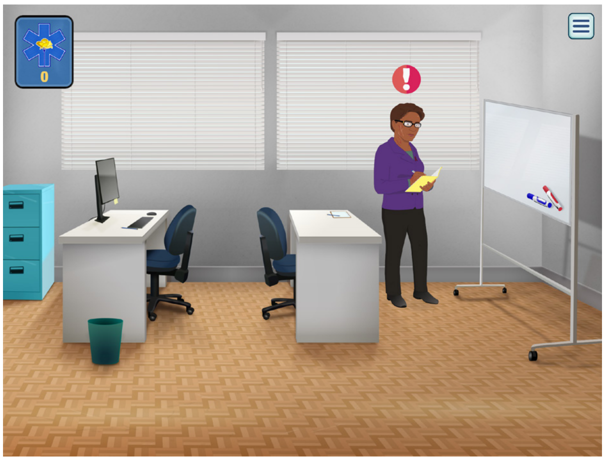

Players can access the Detect and Protect **Badge** here.

12

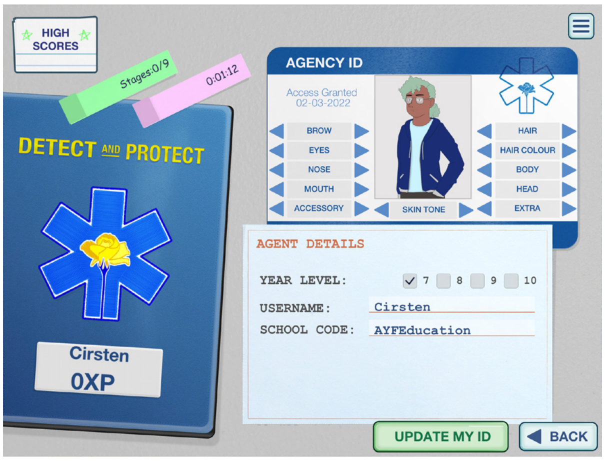


Navigate your points and progress by opening Badge

- High Scores
- Stage completion, out of 9
- Play time
- Personalise Agency ID - character appearance

**Note:** Throughout the game, the player is awarded **experience points** for input in Quizzes and Match the Facts activities.

Progress and score can be checked in the **Badge**.

13

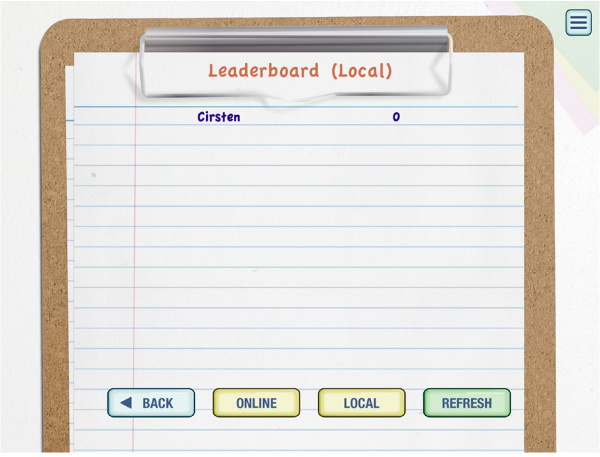


Leader board (Local)

- Local refers to the high scores leader board for that specific device (isolated to that iPad or computer).

14

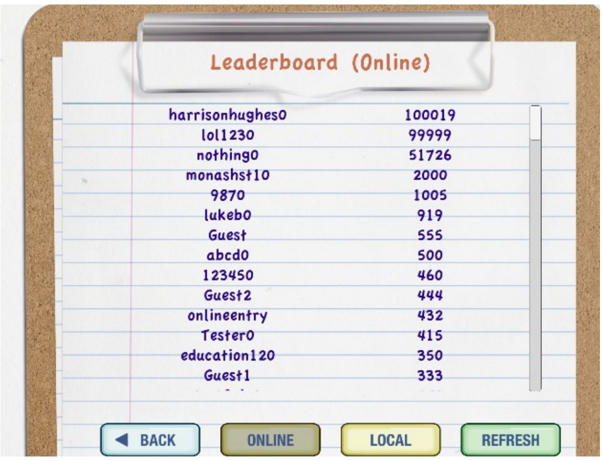


Leader board (Online)

- Online refers to a global leader board of high scores, capturing everyone that has played MIApp.

15

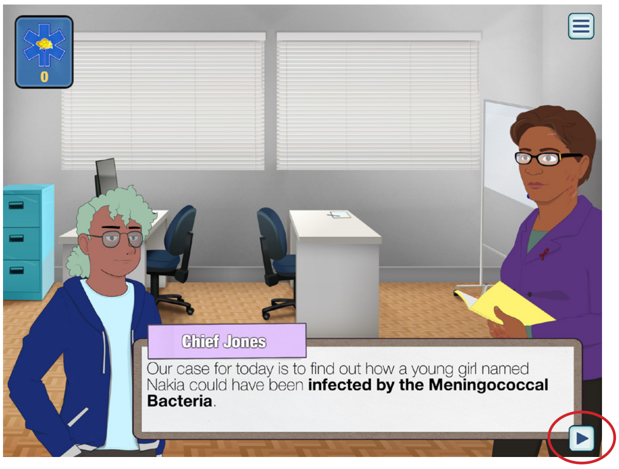


Player will first meet with Chief Jones and be introduced to investigating the case of how a girl named Nakia could have been infected with Meningococcal Bacteria.

Arrows (shown in red circle above) will prompt the player to progress to next dialogue/ screen.

16

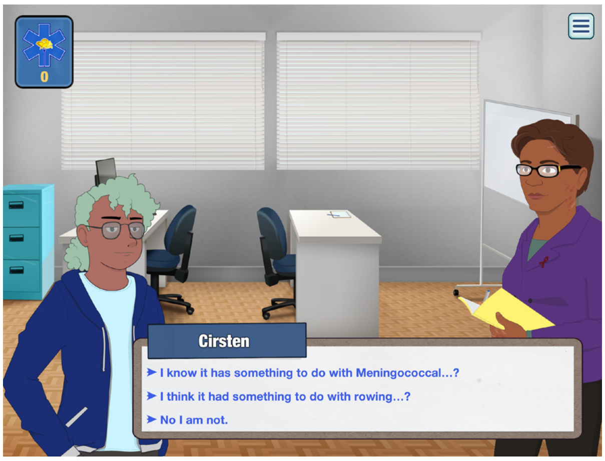


Player is introduced to the Amanda Young Foundation and asked a question.

- Response can be any of three (3) options.

17

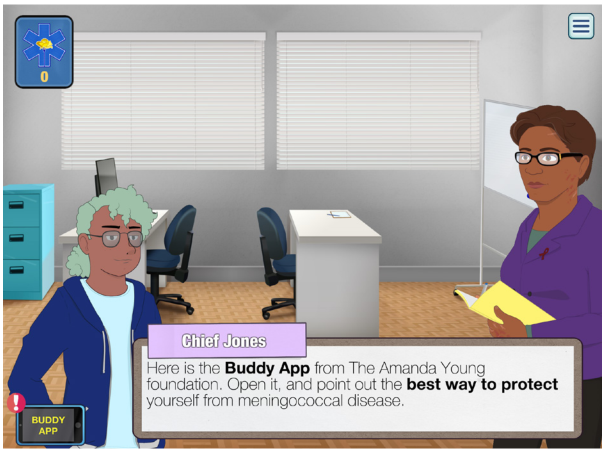


The Buddy App

Buddy is the Amanda Young Foundation mascot.

The **Buddy App** is used as a tool by player throughout the game to check facts on meningococcal disease, and store **Extras** – collectible items.

Player is guided to **open** the **Buddy App** (refer to red exclamation mark).

18

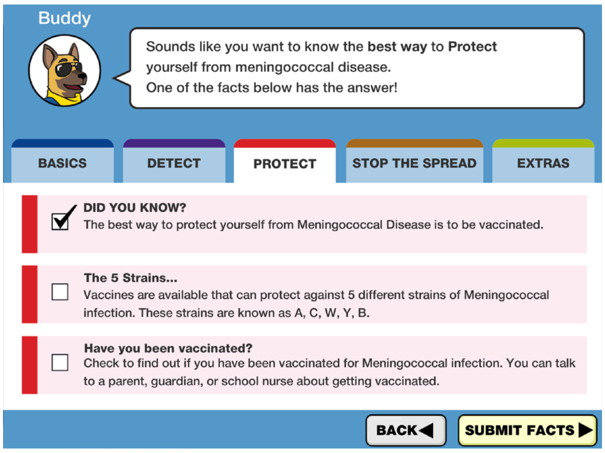


Inside Buddy App:

**PROTECT**

- Select the best way to protect yourself form meningococcal disease

**SUBMIT FACTS:** *Did You Know? The best way to protect yourself from Meningococcal Disease is to be vaccinated*

19

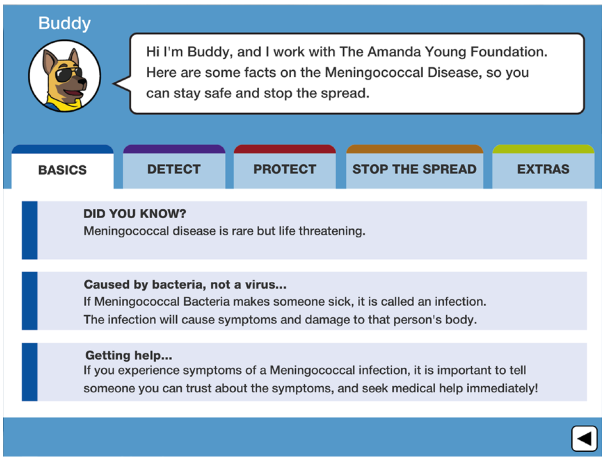


**BASICS:** Facts – Cause and seeking help

20

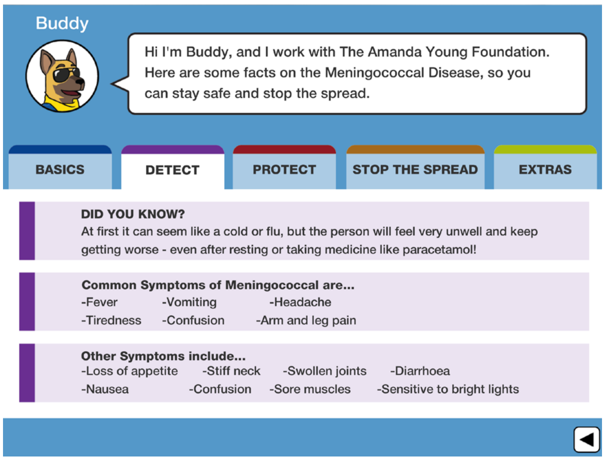


**DETECT:** Facts – Symptoms of meningococcal disease

20

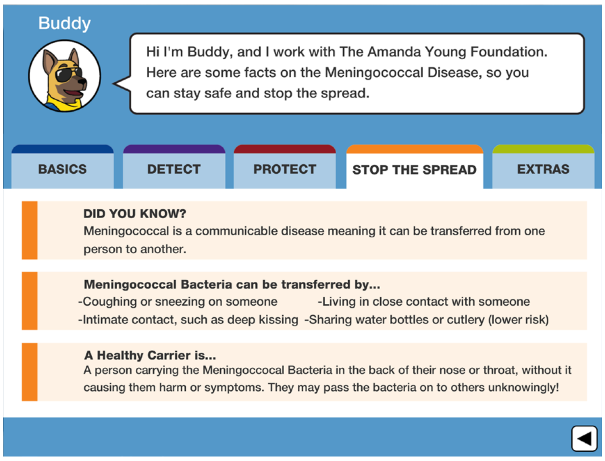


**STOP THE SPREAD:** Facts - Preventing meningococcal disease infection

21

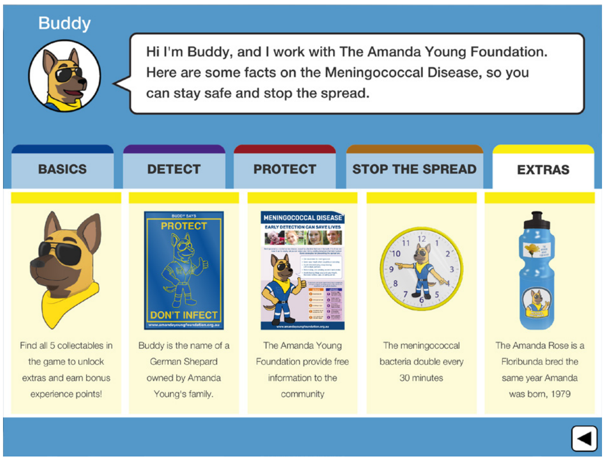


**EXTRAS**

- Find all 5 collectibles (above) with 'Buddy' on them throughout the game, to unlock extras and earn bonus experience points

22

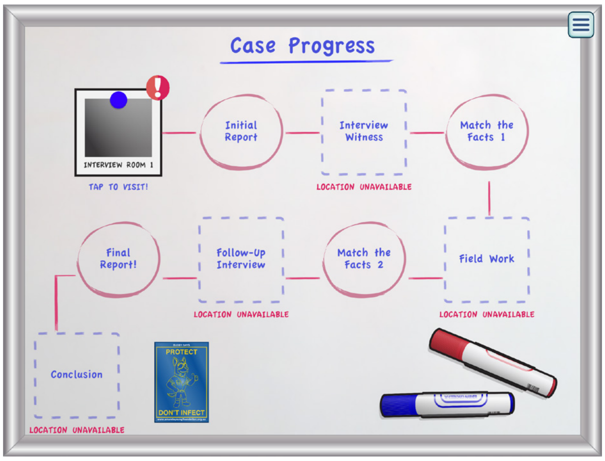


**Case Progress**

- Players can follow the progress of their game through each stage of the investigation
- Access by clicking on the whiteboard in the agency office

23

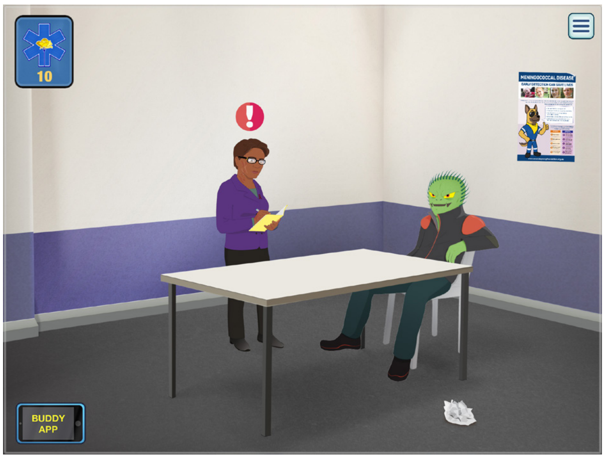


Player is introduced to the character MB (Meningococcal Bacteria), in the Interview Room.

24

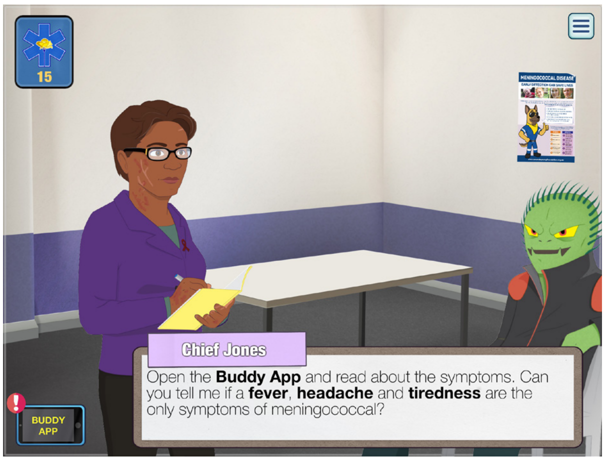


Follow the prompts to the Buddy App and review the symptoms of meningococcal.

When asked to open Buddy App again and point out all symptoms, tick all three (3) answers (for full points).

25

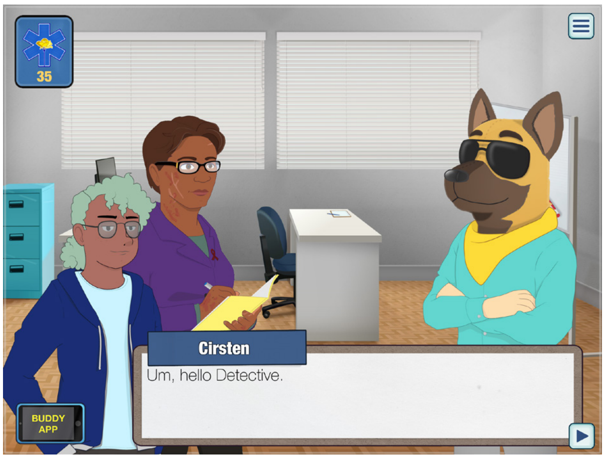


Player is introduced to character Detective Pham.

26

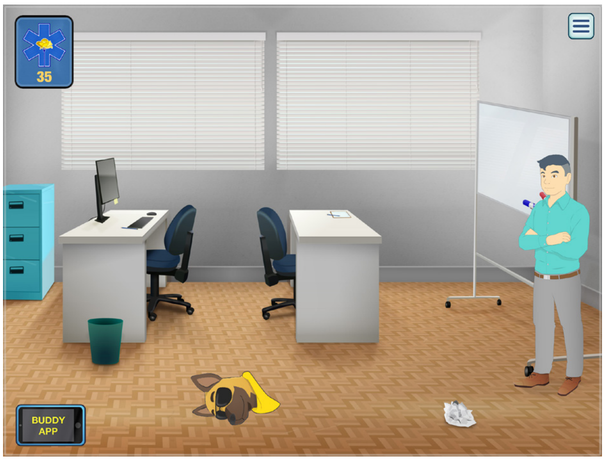


Tap on the mascot Buddy head to get the collectible.

27

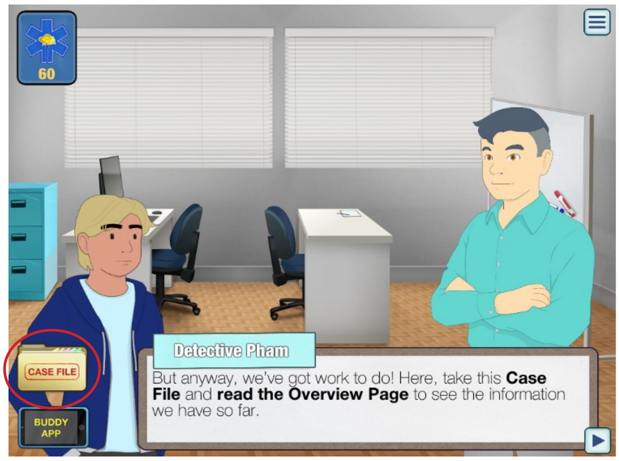


Player is shown how to access the **Case File** and read the **Overview Page** and all information collected in the case so far.

Tap the **Case File** icon to open.

28

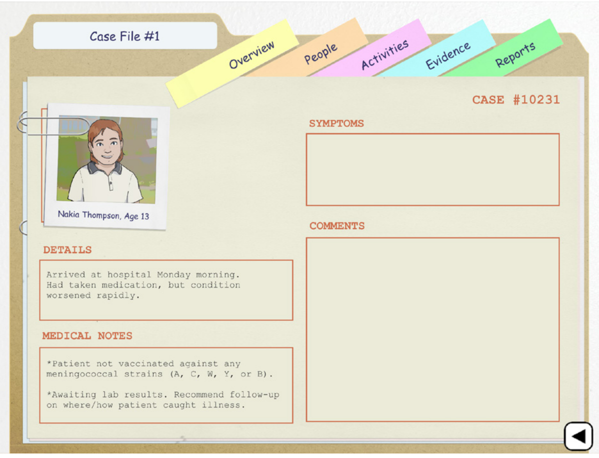


**Overview Page**

- Summary of patient Nakia

29

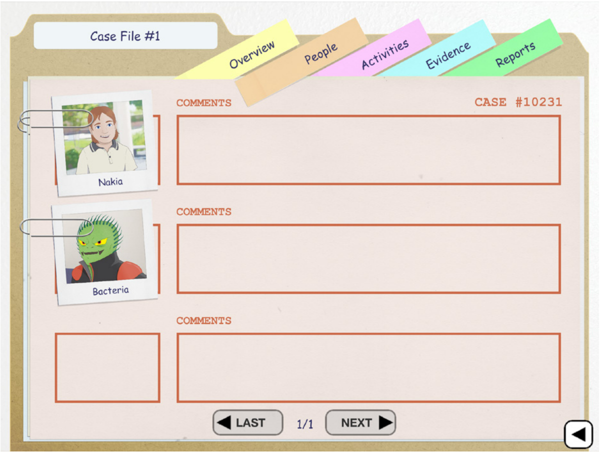


**People**

- List of characters interviewed

30

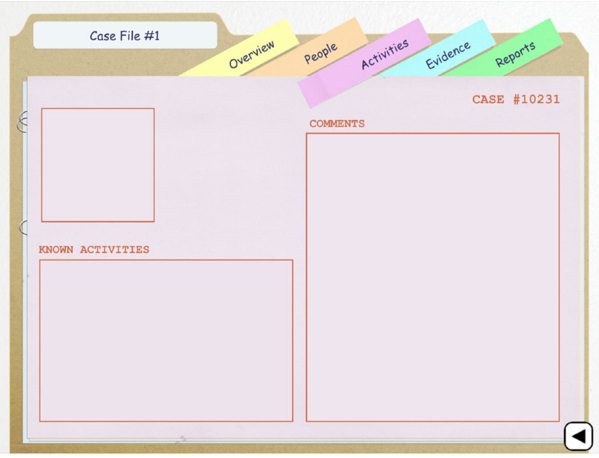


**Activities**

- Known activities will be recorded here as investigation progresses

31

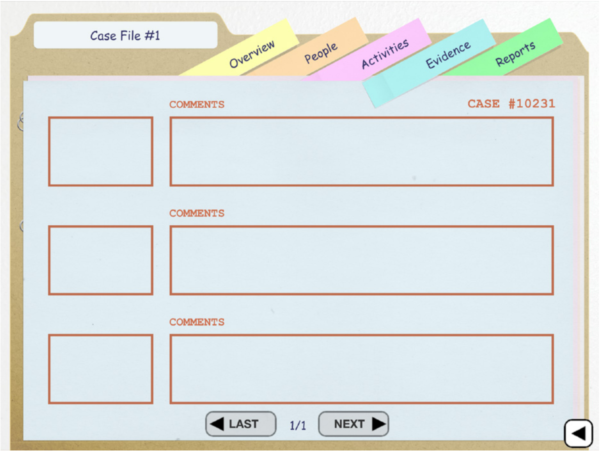


**Evidence**

- List of all evidence collected will be stored here as investigation progresses

32

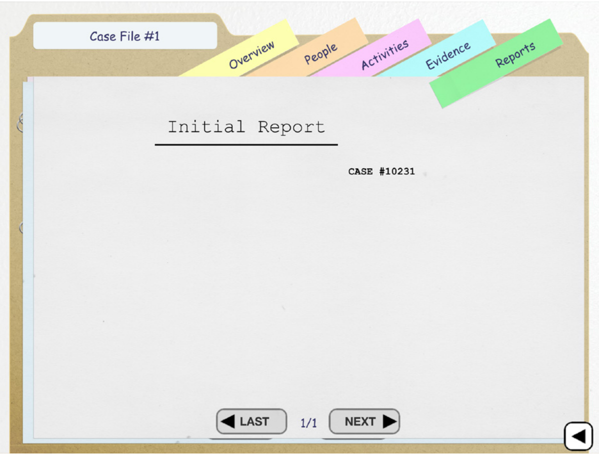


**Reports**

- Initial Report results

33

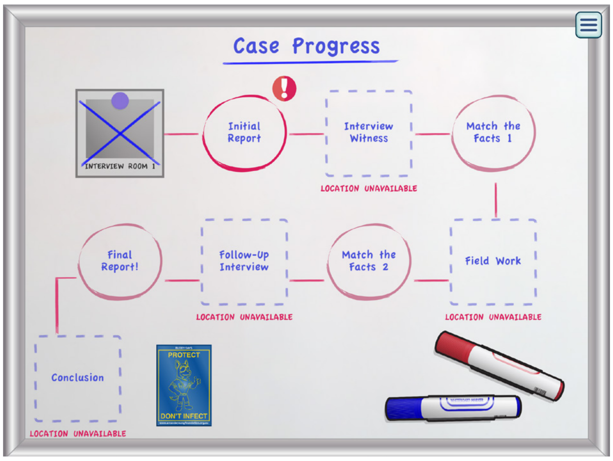


Complete the Initial Report

34

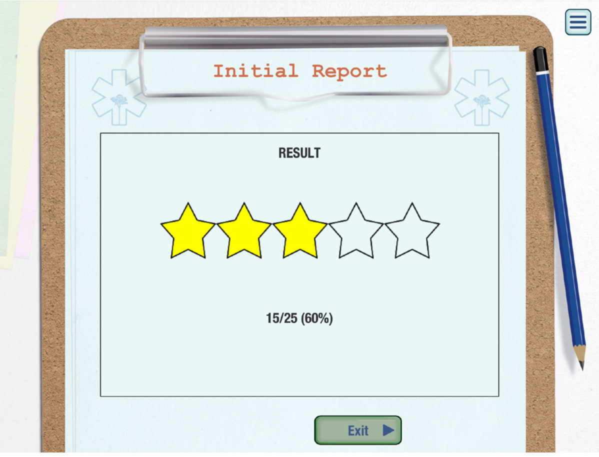


## INITIAL REPORT (QUIZ 1) – Answers

1 - True
2 - Bad headache, vomiting, feeling tired
3 - True
4 - True
5 - False

35

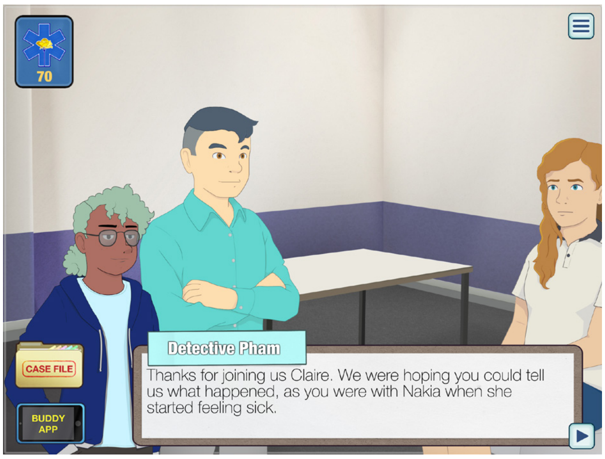


Player is introduced to Nakia's friend, Claire, in the Interview Room.

Interview Claire for any information to help see what could have caused Nakia to get sick.

36

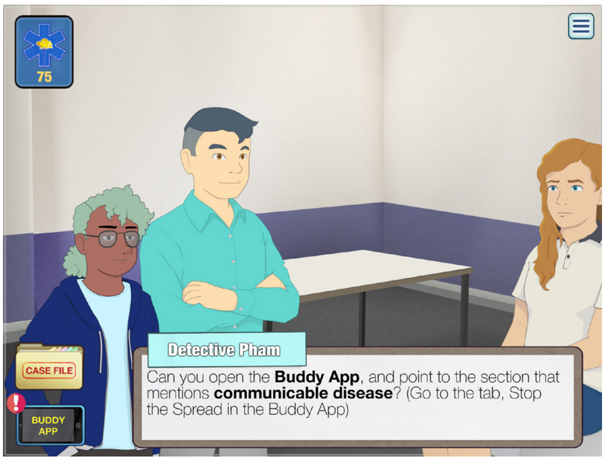


Player is prompted to open the Buddy App once again – Stop the Spread

37

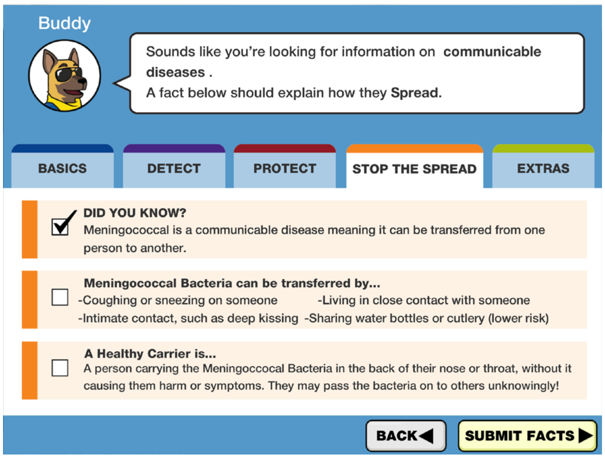


Tick all three (3) boxes and click Submit Facts.

38

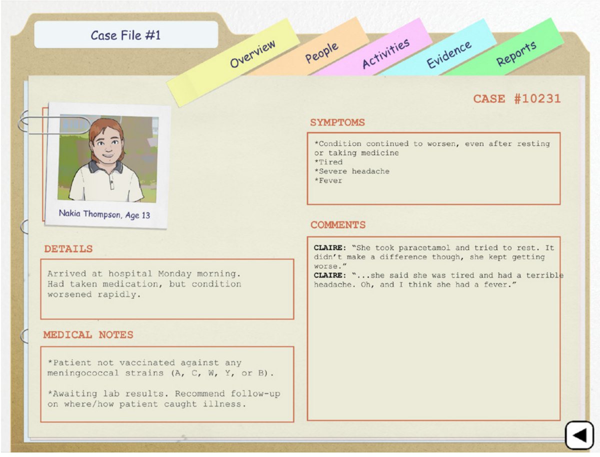


Select the Case File as the investigation progresses. This will be updated as more information is discovered.

39

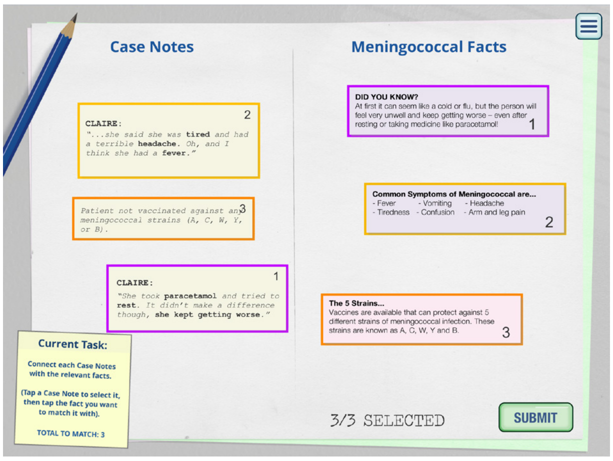


**MATCH THE FACTS 1 – Answers**

- Connect each Case Notes with the relevant facts (total to match: 3)

40

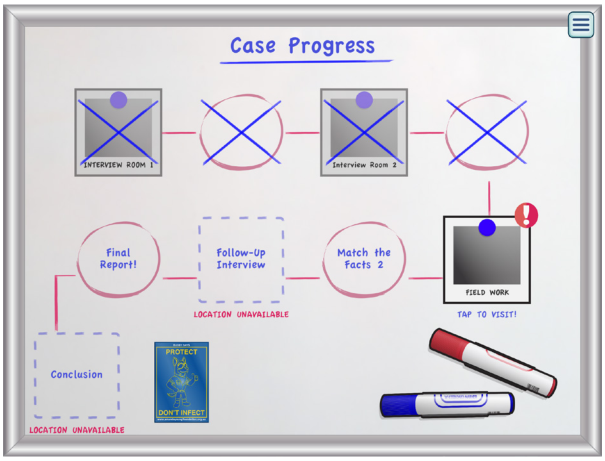


Field Work

41

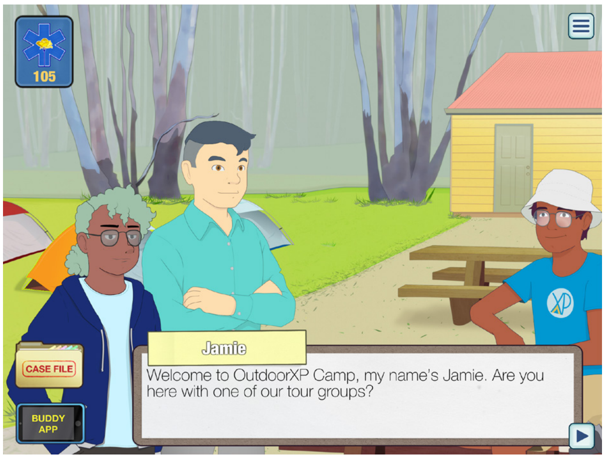


Player is introduced to character at camp, Jamie.

42

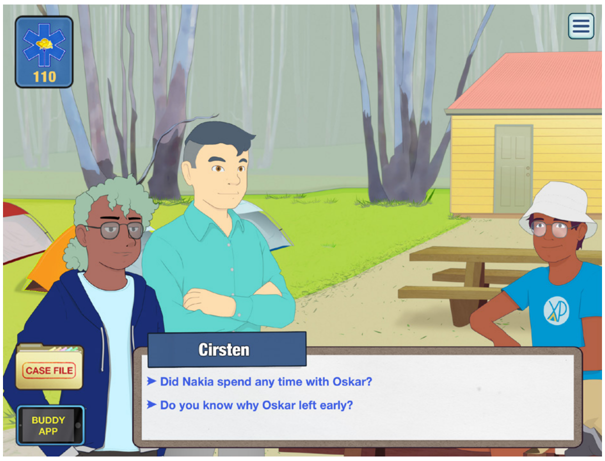


43

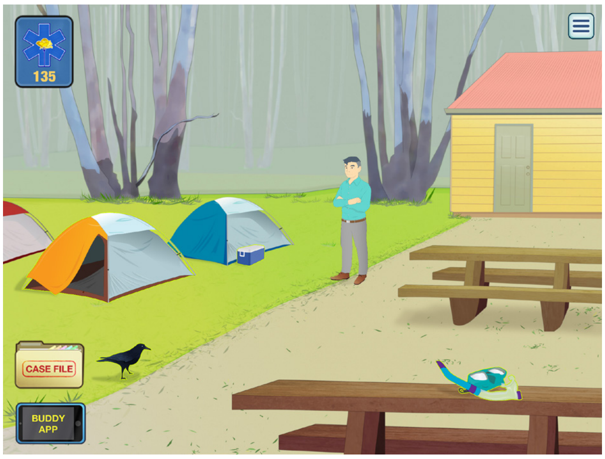


**Look for evidence** - explore the objects around the campsite to see if there was a way the meningococcal bacteria could have been transferred during activities.

44

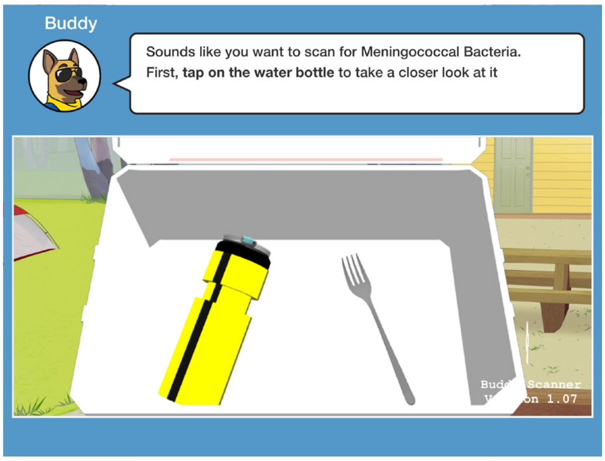


Use the Buddy App Scanner to scan objects.

Tap the Esky to observe the objects inside.

45

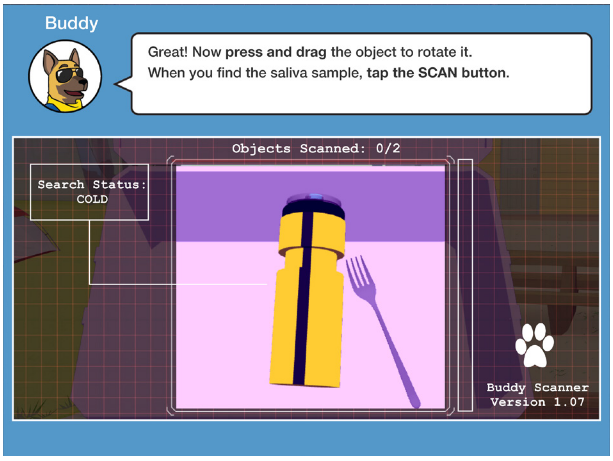


Rotate the Water Bottle and Fork to scan the saliva samples on both objects.

46

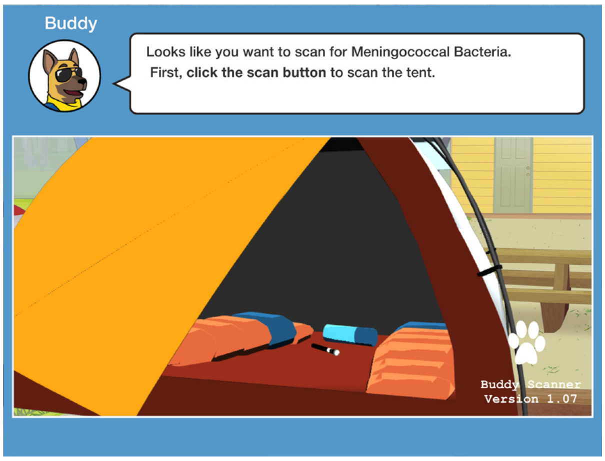


Tap on the tent, and select the SCAN button.

47

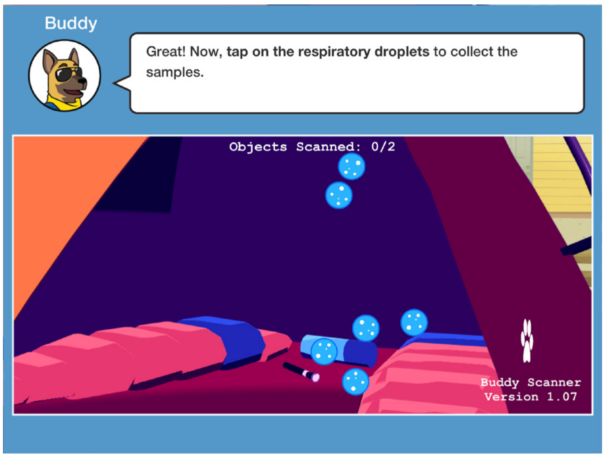


Tap on the respiratory droplets to collect samples.
Total objects to scan: 6

48

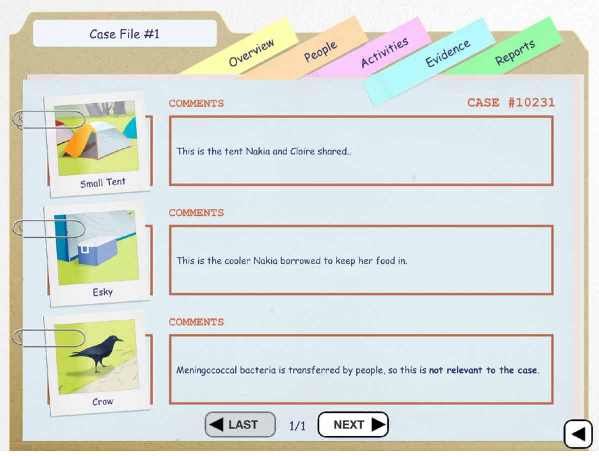


Evidence is updated in the Case File.

49

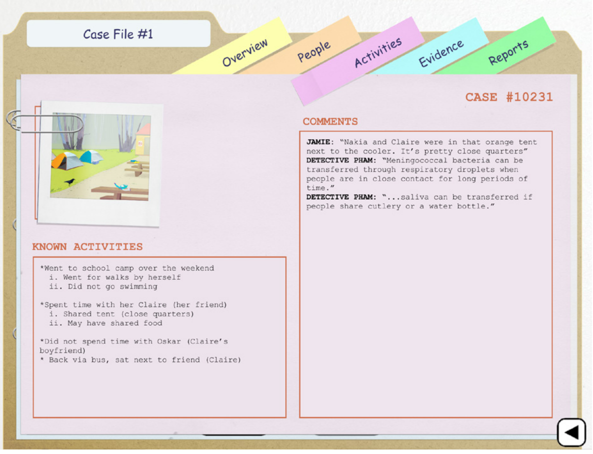


Activities details are updated in the Case File.

50

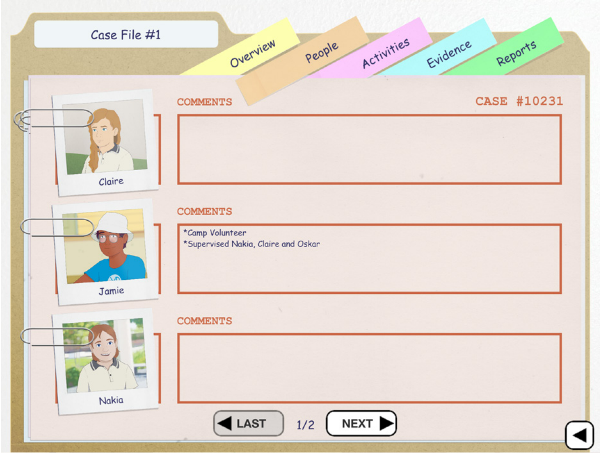


Record of People is updated in the Case File.

51

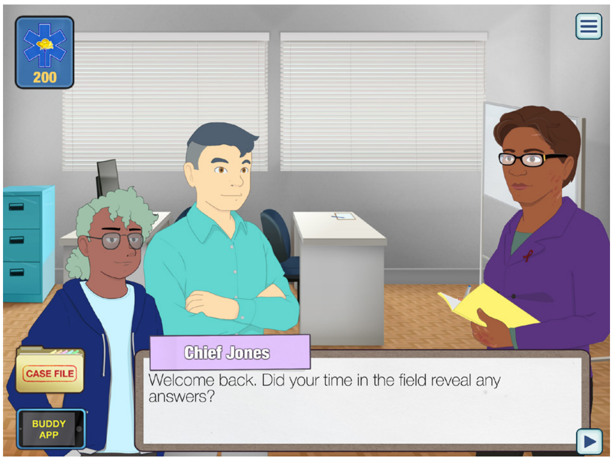


Player is taken back to the agency office to update Chief Jones on latest case developments.

52

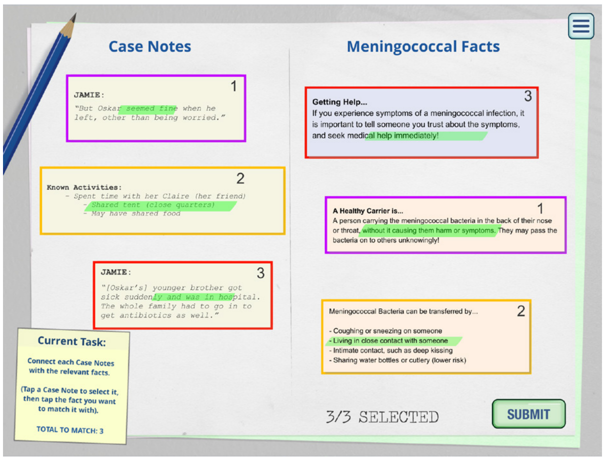


**MATCH THE FACTS 2 – Answers**

- Connect each Case Notes with the relevant facts (total to match: 3)

53

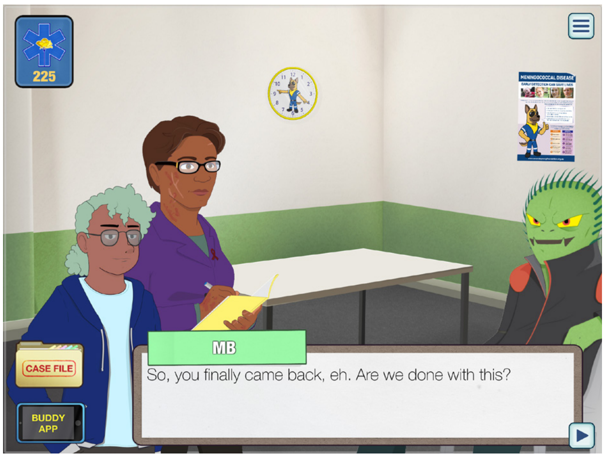


Final interview with MB

54

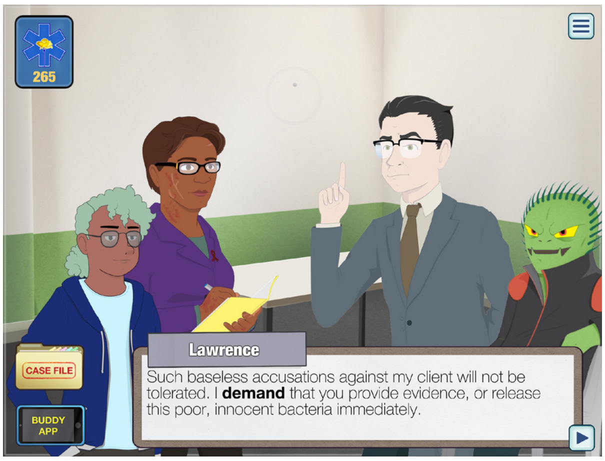


Player introduced to Attorney Lawrence.

55

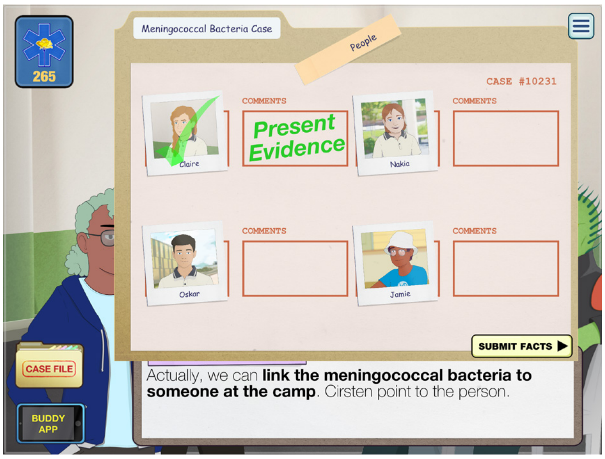


Player is prompted to select which People the case has Present Evidence for – Claire

56

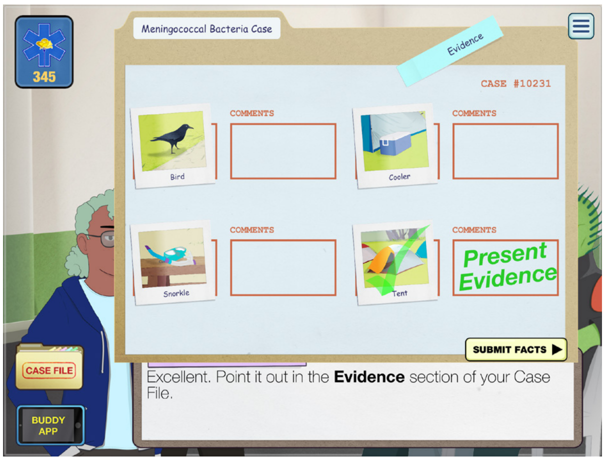


57

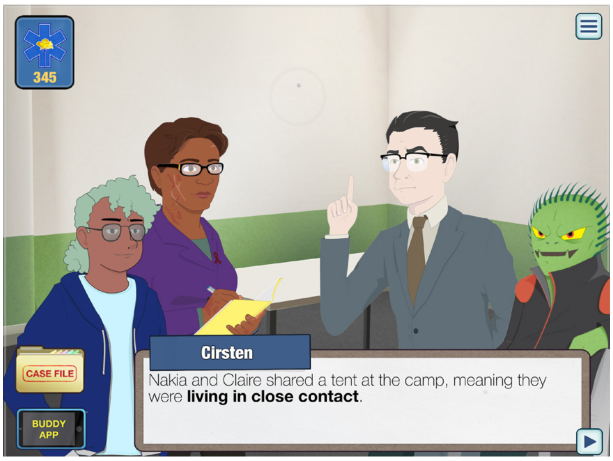


Player is prompted to select which People the case has Present Evidence for – Tent and Cooler

Meningococcal bacteria likely to be transferred from Claire to Nakia – sharing a tent at camp (close contacts).
This confined space could lead to transfer of bacteria through respiratory droplets. Oskar, Claire's boyfriend, likely spread to Claire via intimate contact, such as deep kissing.

Case has now been solved. Player is prompted to complete the Final Report.

58

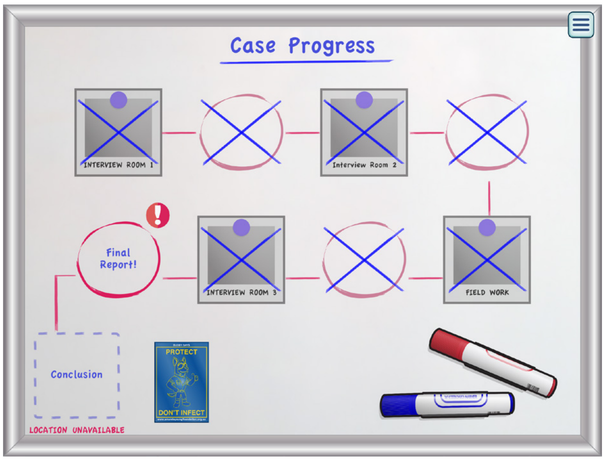


59

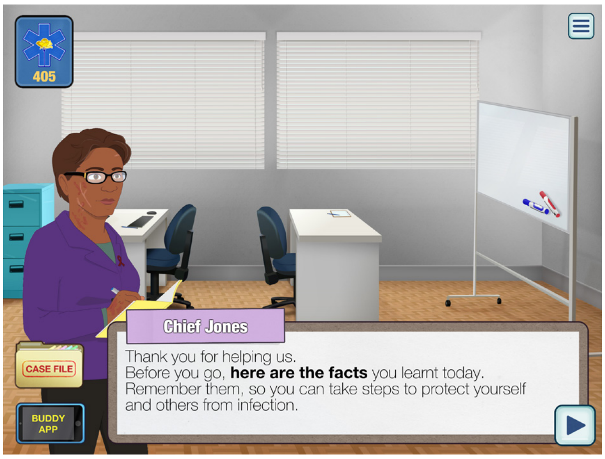


## FINAL REPORT (QUIZ 2) – Answers

1 - True
2 - Bad headache, fever, feeling tired
3 - True
4 - Sharing cutlery or a water bottle, living in close contact with someone, deep kissing
5 - True
6 - All
7 - False
8 - True

Game is finished at completion of case with the Detect and Protect agency.

- Player is given final score
- Scores can be compared in the **Badge – High Scores**

60

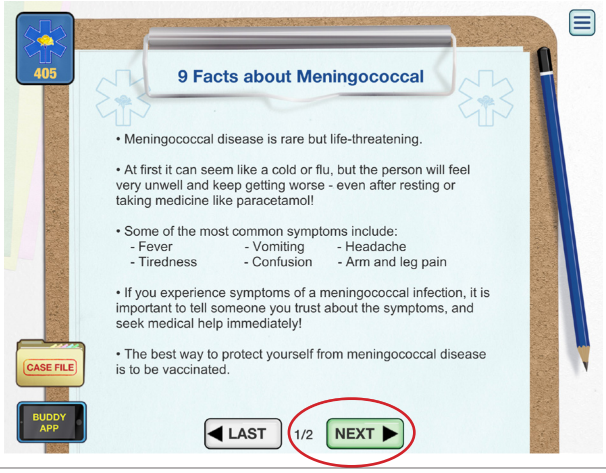


9 Facts about Meningococcal

At completion of MIApp, player will be taken to a screen with a clipboard, displaying the nine key facts learnt throughout the game.

Students are encouraged to record these to take steps toward protecting themselves and others against infection

61

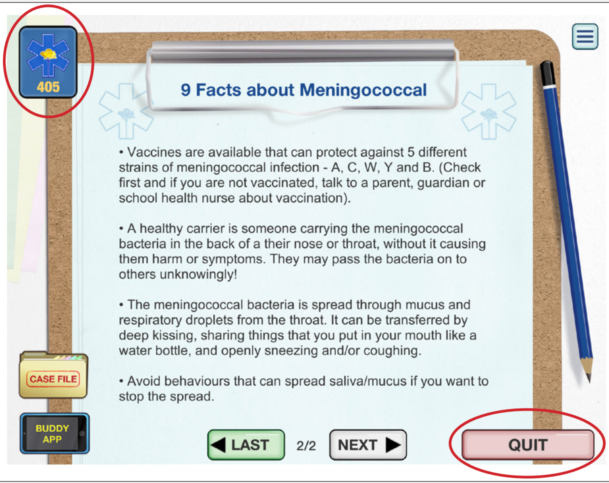


**End of game - Quit**
