# Supplementary material for: Evaluating the Efficacy of a Serious Game to Deliver Health Education About Invasive Meningococcal Disease: Clustered Randomized Controlled Equivalence Trial
Source: JMIR Serious Games. 2025 Feb 11;13:e60755. doi: 10.2196/60755 (PMC11862768; doi:10.2196/60755)
Supplement: Supplementary file 5 [file games_v13i1e60755_app1.docx]

MIAPP Post-Study Questionnaire

Start of Block: Pre-study

|  |
| --- |

Q1 
Thank you for participating! We are now going to answer some questions about MD again, to see what you have learned today.

So we can match these responses to the ones you gave before the lesson, please enter your unique ID

________________________________________________________________

|  |
| --- |

Q2 Please enter your school code

________________________________________________________________

| Page Break |  |
| --- | --- |

Q8 Please answer TRUE or FALSE for this block of questions. It is fine if you still don't know the answer - rather than guessing if you are not sure you can choose 'I'm not sure'

|  | TRUE (1) | FALSE (2) | I'M NOT SURE (3) |
| --- | --- | --- | --- |
| Meningococcal disease is common (1) |  |  |  |
| Someone with Meningococcal disease can die from it (2) |  |  |  |
| Early symptoms of Meningococcal disease are similar to a cold or flu (3) |  |  |  |
| There is a vaccine for Meningococcal disease (4) |  |  |  |
| Taking paracetamol and resting will cure Meningococcal disease (5) |  |  |  |
| Someone with Meningococcal disease will be given antibiotics (6) |  |  |  |
| Meningococcal disease can be spread by kissing (7) |  |  |  |
| The best way to protect yourself from Meningococcal disease is to be vaccinated (8) |  |  |  |
| Meningococcal disease is caused by a virus (9) |  |  |  |
| Meningococcal disease can be spread by blood (10) |  |  |  |
| A person can carry Meningococcal in their body without becoming sick (11) |  |  |  |

Q9 Which of the
following are symptoms of Meningococcal Disease? (choose all that apply)

- Fever  (1) 

- Rash  (2) 

- Numbness  (3) 

- Pain in the arms and/or legs  (4) 

- Eye twitching  (5) 

- Cramps  (6) 

- Confusion  (7) 

- Constipation  (8) 

- I don't know any of the symptoms of meningococcal disease  (9) 

Q10 If you thought you or someone
you know might have meningococcal disease, how long should you wait before
telling someone and getting help? (choose only ONE answer)

- You should tell someone within a week  (1) 

- You should tell someone within a day  (2) 

- You should tell someone within a few hours  (3) 

- You should tell someone immediately  (4) 

- I don't know  (5) 

Q11 Which of these
activities could best help to prevent the spread of meningococcal disease? (choose all that apply)

- Making sure you don't eat undercooked food  (1) 

- Avoiding sharing things you put in your mouth e.g. water bottles  (2) 

- Using insect repellent  (3) 

- Making sure you don't drink tap water on holidays  (4) 

- Washing your hands thoroughly after you use the toilet  (5) 

- I don't know  (6) 

Q12 Which of these people
is most likely to pass on meningococcal disease? (choose only ONE answer)

- Some who appears to be very sick  (1) 

- Someone who appears to be healthy  (2) 

- I don't know  (3) 

Q13 Where in the body is meningococcal most likely to live? (choose only ONE answer)

- Under the arm pits  (1) 

- In the back of the nose and throat  (2) 

- In the ears  (3) 

- In the stomach  (4) 

- I don't know  (5) 

| Page Break |  |
| --- | --- |

Q14 Did you play MIAPP today, or did you receive an in-class presentation?

- MIAPP  (1) 

- In-class presentation  (2) 

Skip To: End of Survey If Did you play MIAPP today, or did you receive an in-class presentation? = In-class presentation

| Page Break |  |
| --- | --- |

Q15 These questions are asking you opinion about MIAPP. Please answer
these as honestly as you can. Remember, there are no right or wrong
answers; we are just interested to hear what you think

|  | Strongly disagree (1) | Somewhat disagree (2) | Neither agree nor disagree (3) | Somewhat agree (4) | Strongly agree (5) |
| --- | --- | --- | --- | --- | --- |
| I would like to use educational games like this more often. (1) |  |  |  |  |  |
| Finding things in MIAPP was too hard. (2) |  |  |  |  |  |
| It was clear when I was playing what I was supposed to do next (3) |  |  |  |  |  |
| I needed help from my teacher to be able to use MIAPP. (4) |  |  |  |  |  |
| MIAPP was more enjoyable than a presentation (5) |  |  |  |  |  |
| I thought the features on the screen of MIAPP were confusing (6) |  |  |  |  |  |
| I thought MIAPP was easy to use. (7) |  |  |  |  |  |
| I found using MIAPP a bit overwhelming. (8) |  |  |  |  |  |
| I felt very confident using MIAPP. (9) |  |  |  |  |  |
| I needed to learn a lot more before I could understand MIAPP. (10) |  |  |  |  |  |

End of Block: Pre-study
